# Learned Conformational Space and Pharmacophore Into Molecular Foundational Model

**DOI:** 10.1002/advs.202513556

**Published:** 2026-01-04

**Authors:** Lin Wang, Yifan Wu, Hao Luo, Minglong Liang, Yihang Zhou, Cheng Chen, Chris Liu, Jun Zhang, Yang Zhang

**Affiliations:** ^1^ Center for AI and Computational Biology Institute of Systems Medicine Chinese Academy of Medical Sciences Suzhou China; ^2^ State Key Laboratory of Common Mechanism Research for Major Diseases Institute of Systems Medicine Chinese Academy of Medical Sciences Suzhou China; ^3^ Cancer Science Institute of Singapore National University of Singapore Singapore Singapore; ^4^ DeepMed Technology (Suzhou) Co., Ltd Suzhou China; ^5^ Department of Computer Science School of Computing National University of Singapore Singapore Singapore; ^6^ Department of Biochemistry Yong Loo Lin School of Medicine National University of Singapore Singapore Singapore

**Keywords:** artificial intelligence, conformational space, drug discovery, molecular foundational model, molecular generation

## Abstract

The emergence of large‐scale chemical pre‐trained models has significantly advanced our ability to capture complex relationships between molecular structures and their functions. Despite the growing interest in molecular foundational model that provide versatile representations and support molecular optimization for downstream tasks, few efforts have integrated explicit chemical knowledge—such as conformational and pharmacophore information—into pre‐training. Given the highly dynamic nature of small molecules in solution, their conformational changes upon target binding, and the critical role of pharmacophore complementarity, it is essential to incorporate these factors into molecular foundational modeling. Here, we present a molecular foundational model that integrates conformational‐space and pharmacophore‐similarity projections during pre‐training to regularize the representation space. The model adopts an Ouroboros‐like architecture, where the molecular graphs are encoded into 1D representation vectors via a graph neural network and subsequently reconstructed back into SMILES sequences through an autoregressive Transformer module. This dual‐module design establishes a flexible and extensible framework for both representation learning and molecular generation within a unified latent space. Extensive experiments demonstrate that our model effectively addresses a variety of practical chemical challenges, including similarity‐based virtual screening, targeted poly‐pharmacology design, chemical property prediction, and directed molecular optimization.

## Graphical abstract

The Ouroboros model introduces two orthogonal modules within a unified framework that independently learn molecular representations and generate chemical structures. This design enables flexible optimization strategies for each module and faithful structure reconstruction without prompts or noise. By integrating conformational space and pharmacophore projections, Ouroboros enables versatile applications ranging from molecular property prediction to targeted poly‐pharmacology and structure optimization.

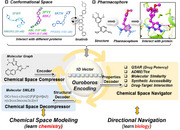

## 1 Introduction

Artificial intelligence (AI) is already being applied to a wide range of fields in chemical research, including materials design, drug discovery, and computational biology [1]. Data‐driven approaches have revolutionized how people predict the properties, interactions, folding, and assembly of molecules [2, 3, 4, 5]. The community efforts toward this have not only made intensive use of many techniques derived from natural language processing and computer vision [6, 7, 8], but also developed graphical neural networks better suited to molecular structures [9, 10]. Most recently, growing interests have been shown in general foundational models for small molecules [11, 12, 13], such work aims to build a foundational model to improve their performance in downstream property prediction.

Capturing the meaningful representation space of chemical structure is the general principle of molecular foundational models, just as a deep neural network to learn the underlying data structure [14, 15]. For instance, considerable progress has been recently achieved in leveraging deep neural networks to learn interpretable molecular representations [16], which can be of critical help in molecular property prediction and similarity virtual screening [17, 18, 19, 20, 21]. Various pre‐training strategies incorporate different chemical knowledge for the chemical pre‐trained models to facilitate their application in a variety of downstream tasks [22]. Self‐supervised pre‐training strategy is designed to understand different chemical representation formats (SMILES, Graph, InChI, molecular fingerprints) [17, 20, 23, 24, 25, 26, 27], while supervised pre‐training is designed to integrate the relationship between the chemical structure and their multiple modalities, functions, or properties [13, 28, 29, 30, 31, 32].

Emergence of generative pre‐training has enabled generative capabilities for molecular foundational models, by generating both molecular structure and chemical properties [13, 29, 33], such work can leverage labeled chemical datasets to train a unified representation and generative foundational model. However, labeled high‐quality experimental datasets are so scarce that people have to generate large amounts of simple labels through cheminformatics tools, which prevents models from acquiring complex chemical knowledge from pre‐training. Additionally, generative pre‐training models need to adopt a chemical language that can be tokenized, which prevents model from learning representations through molecular graphs, although some studies have shown that molecular graph is an outperformance algorithm for representation learning [34, 35].

In this work, we proposed two orthogonal modules in a single model responsible for translating molecular structure (graph) to 1D representation vector and from a representation vector to molecular SMILES (chemical language), which allows us to train representation learning and molecular generation independently, enabling us to select appropriate neural network, dataset, and training strategy for each module. Unlike other molecular generation frameworks, the reconstruction model requires no prompt or noise input, instead faithfully reconstructing chemical structures in the representation space. The orthogonal framework allows our model (named Ouroboros) can be used as a fundamental module for any pharmaceutical small molecule predictor and make the new model capable of optimizing the structure of small molecules and even *de novo* molecular design. The Ouroboros has demonstrated its representational capabilities not only in similarity virtual screening and targeted poly‐pharmacology drug design, but also in property modeling for a wide range of pharmacokinetic and toxicological properties, which can be applied to further molecular structure optimization.

## 2 Results

### 2.1 The Orthogonal Architecture of Representation and Reconstruction Modules

#### 2.1.1 Learned Conformational Space and Pharmacophore by Representation Learning

Some previous studies have highlighted the importance of pharmacophores in molecular representation [36, 37]; however, they use pharmacophores as input features to the model rather than teaching the model learn how to construct pharmacophores and compare them. In this work, pharmacophore similarity was employed in representation learning to facilitate the model to learn the relationship in molecular shape and properties between different 2D structures, allowing to perceive the intrinsic relevance between different chemical scaffolds. Furthermore, we introduced systematically searched molecular conformational spaces to compare their pharmacophore similarity, this allows our model to perceive the dynamic behavior of the molecules, which is consistent with the knowledge that molecules bind to different targets in different conformations.

In Ouroboros, the representation module creates a structured representation space to interpret chemical molecular structures, with the assumption that molecules with similar chemical structures or pharmacological properties are positioned in proximity within the representation space. Previously, we demonstrated that incorporating conformational space similarity yields a generic molecular representation for ligand‐based drug discovery [19]. To improve the performance of molecular representation learning, here we introduce two kinds of inter‐molecular similarities, including an extended CSPS with enhanced space searching and molecular fingerprint similarity (see **Methods**).

**FIGURE 1:**
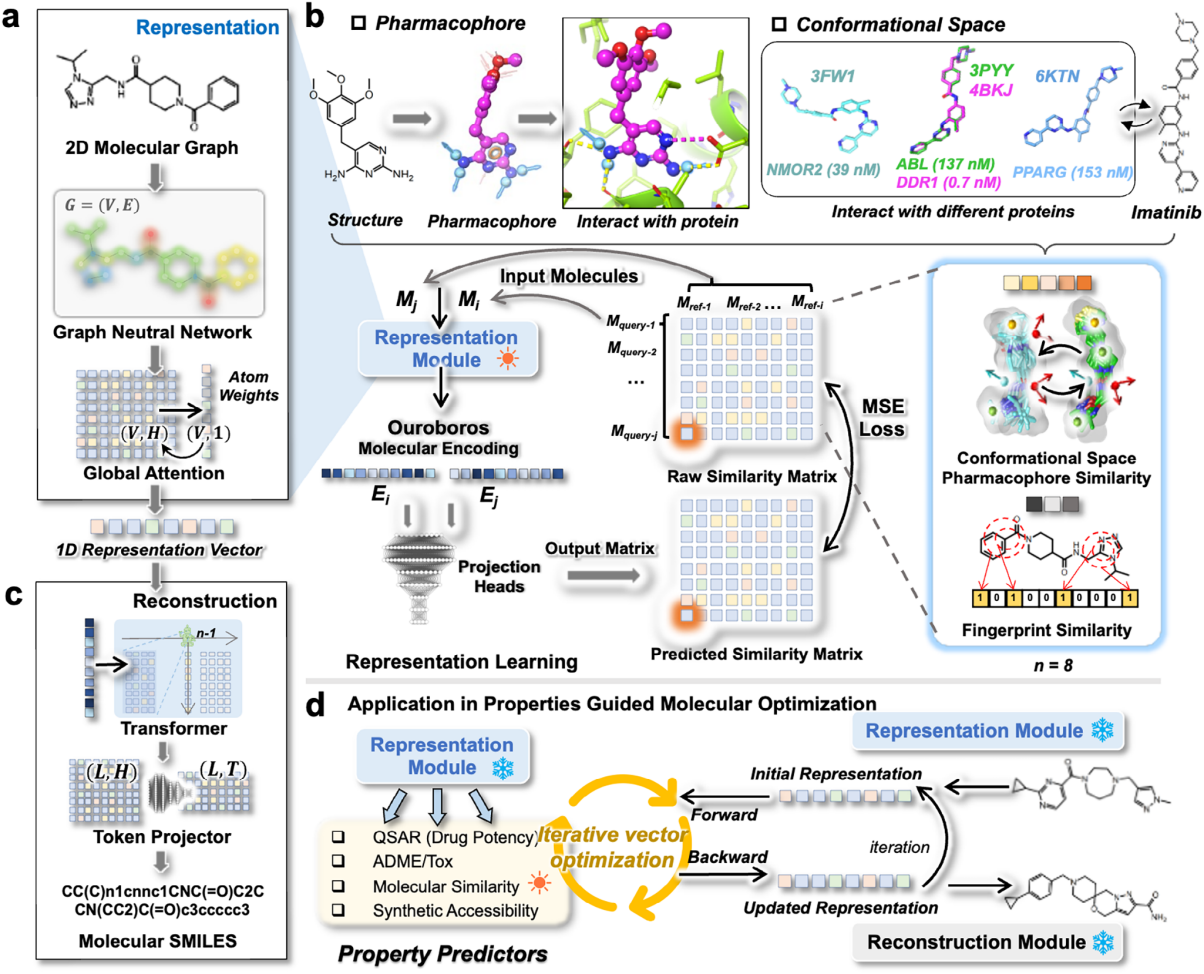
The motivation and architecture of Ouroboros molecular foundational model. (a) The representation module converts molecular graphs into 1D representation vectors by a graph neural network (GNN) and atom‐wise attention mechanism. (b) The motivation for representation learning for Ouroboros. The pharmacophore and conformational space of the small molecules demonstrate the manner in which it carries out its pharmacological function. In the pharmacophore view, hydrogen bond donors are labeled as sky blue arrows, hydrogen bond acceptors are labeled as red arrows, aromatic rings are labeled as orange circle/sphere, and hydrophobic groups are labeled as green sphere. In the ligand‐pocket binding pose, hydrogen bond is shown as yellow dashed line and salt bridge is shown as magenta dashed line. The PDB code for imatinib's experimental ligand structure is labeled next to the molecular conformation. The “*i*” refers to the index of reference molecule and “*j*” to index of query molecule. (c) The reconstruction module of Ouroboros. This module transforms these 1D representation vectors into SMILES representations, utilizing an auto‐regressive generative model by Transformer decoder. (d) Applying the Ouroboros to specific computational biology/chemistry scenarios. With the participation of datasets and other entity representations relevant to specific scenarios, the property predictor can be trained to project the 1D representation vectors to molecular properties through a multilayer perceptron (MLP). With a gradient‐based optimizer, Ouroboros' representation vectors can be optimized in both forward and backward propagation, while the loss function is constructed with the aforementioned property predictors.

We propose a new similarity learning strategy that projects molecular representations into multiple molecular similarities, aiming to enhance the capacity of the representation module for generic and informative molecular representation. The representation module, which transforms molecular graphs into 1D representation vectors via a graph neural network (Figure 1a), is pre‐trained on a similarity matrix constructed from pairwise similarities between query and reference molecules. The representation module is regularized into a meaningful representation space by Conformational Space Pharmacophore Similarity (CSPS) learning (Figure 1b), which is a large‐scale similarity matrix (4728 × 126 248 × 8, 4.7 billion molecular similarities) constructed from a small number of chemical structures (126 248). The representation learning aims to validate the model generalization capability on benchmark datasets (more than 1.6 million).

During the representation learning, the 1D representations are projected by two external projection heads into fingerprint similarity and CSPS respectively with the training process guided by the Mean Squared Error (MSE) loss (Figure 1b). Figure S1a illustrates how the molecular representation is extracted from molecule graphs, where atoms and chemical bonds in the molecular graphs are represented through a GNN for message passing. The resulting parameterized graph is then processed through a global attention pooling module [38] to construct the molecular representation. Here, a MLP module designed specifically for projecting 1D representation vectors into similarities (Figure S1b). The Spearman correlation on the validation dataset reached convergence after about 20 000 pre‐training steps (Figure S2), finally, all CSPS showed a similar and high performance on the test set (Table S1), i.e., the Spearman correlation exceeded 0.95. The result suggests that the representation module captures the overall rules of the molecular conformational space and pharmacophore, rendering it sufficient to predict the CSPS of the molecule on unseen molecular structures.

#### 2.1.2 Reconstruct Chemical Structures from Molecular Representation Space

In previous studies, chemical language encoders and decoders are typically trained jointly to ensure the accurate reconstruction of molecular representations from hidden layers. However, this coupling presents challenges for applying molecular representation learning in generative AI. One key issue is that the information learned within the intermediate hidden layers of encoder–decoder models often fails to generalize effectively across diverse molecular properties. In this work, we developed a reconstruction module that reconstructs the original SMILES representations of molecules from their encoded 1D vectors using a Transformer‐based architecture (Figure 1). An auto‐regressive transformer [39] module has been trained to convert this 1D representation vector back to the chemical structure in molecular SMILES format (Figure S3), which is trained on a large number of unlabeled chemical structures (48.2 million) and aims to ensure that the model fully explores the representation space to improve molecular generation capability. The model converges rapidly within a single training epoch (Figure S4), this suggests that the auto‐regressive transformer decoder can finalize the reconstruction of SMILES at the token level.

#### 2.1.3 More than 80% Structures are Rapidly Recovered in Validation

To assess its performance, Figure S5 presents the AtomPairs [40] fingerprint similarity between the actual molecular structures and those reconstructed by the Ouroboros in the validation set, where the mean similarity curve quickly stabilizes at an 80% threshold as batch numbers increase, demonstrating the efficiency and robustness of the Ouroboros in molecular structure recovery from 1D representation vectors.

### 2.2 Interpretability of Ouroboros

#### 2.2.1 Atom Attention Weights Visualization

The functional groups in a chemical compound are essential to its properties, hence, taking into account the attentional weights of different atoms in the representation module would facilitate the model to focus on the critical functional groups and captures the varying contributions of different atoms to the overall molecular representation. For atoms of the same element type but with distinct pharmacophore features, the GNN assigns differentiated weights. For example, a nitrogen atom with positive charge is weighted differently from those in amide groups (Figure S7a). Moreover, the model demonstrates heightened attention to bulky hydrophobic groups when they appear, emphasizing their importance in pharmacophore and conformational flexibility.

#### 2.2.2 t‐SNE Analysis of Representation Vectors

Since Ouroboros integrates conformational space and pharmacophore information during representation learning, this may enable it to distinguish molecules with different conformational dynamics or pharmacophores. Hence, we performed dimensionality reduction on Ouroboros representation vectors across three datasets without any fine‐tuning on the task [41]. As shown in Figure S7b, Ouroboros distinguish the molecules with different potential in blood‐brain barrier permeability, eye irritation, and corrosion. The blood‐brain barrier is related to its flexibility and formal charge [42], while eye corrosion and irritation are related to acidity and alkalinity [43], this reveals the underlying mechanism by which Ouroboros can distinguish between them.

#### 2.2.3 Similarity Declines more Rapidly than Validity upon Perturbations

To further assess the ability of the reconstruction module in exploring latent molecular structures within the representation space, we evaluate whether the model can generate valid and diverse molecular structures by introducing stochasticity into the decoding process. As shown in Figure S6, we employed two approaches for randomized decoding: 1) perturbing molecular representation vectors by adding noise based on the representation space distribution and 2) introducing randomness into the token sampling process using the Gumbel‐max technique [44]. As expected, the similarity between the original and decoded molecules decreases with increasing temperature and noise levels. However, the similarity curve declines more rapidly than the validity curve (which measures the proportion of decoded molecules with chemically reasonable valence bonds), indicating that Ouroboros effectively generates novel and diverse molecular structures while maintaining structural validity. These findings highlight again the effectiveness of the proposed reconstruction module in accurately reconstructing molecular structures from their 1D representation vectors.

### 2.3 Applying Representation Module to Similarity‐Based Virtual Screening

#### 2.3.1 Benchmark Task and Datasets

We first examine the representation and generalization abilities of our representation module using zero‐shot similarity assessment, which is designed to identify structurally similar molecules with comparable biological activity. In small molecular drug discovery, similarity assessment can be applied to similarity virtual screening, aiming to identify novel chemical scaffolds by retrieving new molecules that resemble known active compounds. Consequently, the generality and versatility of molecular representation vectors play a critical role in determining the capability of Ouroboros in drug discovery and computational biology.

In this study, the representation module was trained on a limited subset of molecular structures (126 248) while maintaining the ability to generalize across large‐scale benchmark datasets, such as 1 224 678 unique molecules in DUD‐E [45] and 383 893 unique molecules in LIT‐PCBA [46]. Through comparing the cosine similarity between the representation vectors, we can compare Ouroboros with other molecular representation and molecular foundational models, molecular fingerprints, and baseline methods such as experimental structure‐based pharmacophore superimposition, which will show whether Ouroboros can be generalized to large‐scale benchmark datasets.

#### 2.3.2 Similarity Virtual Screening on 117 Single Targets

**FIGURE 2:**
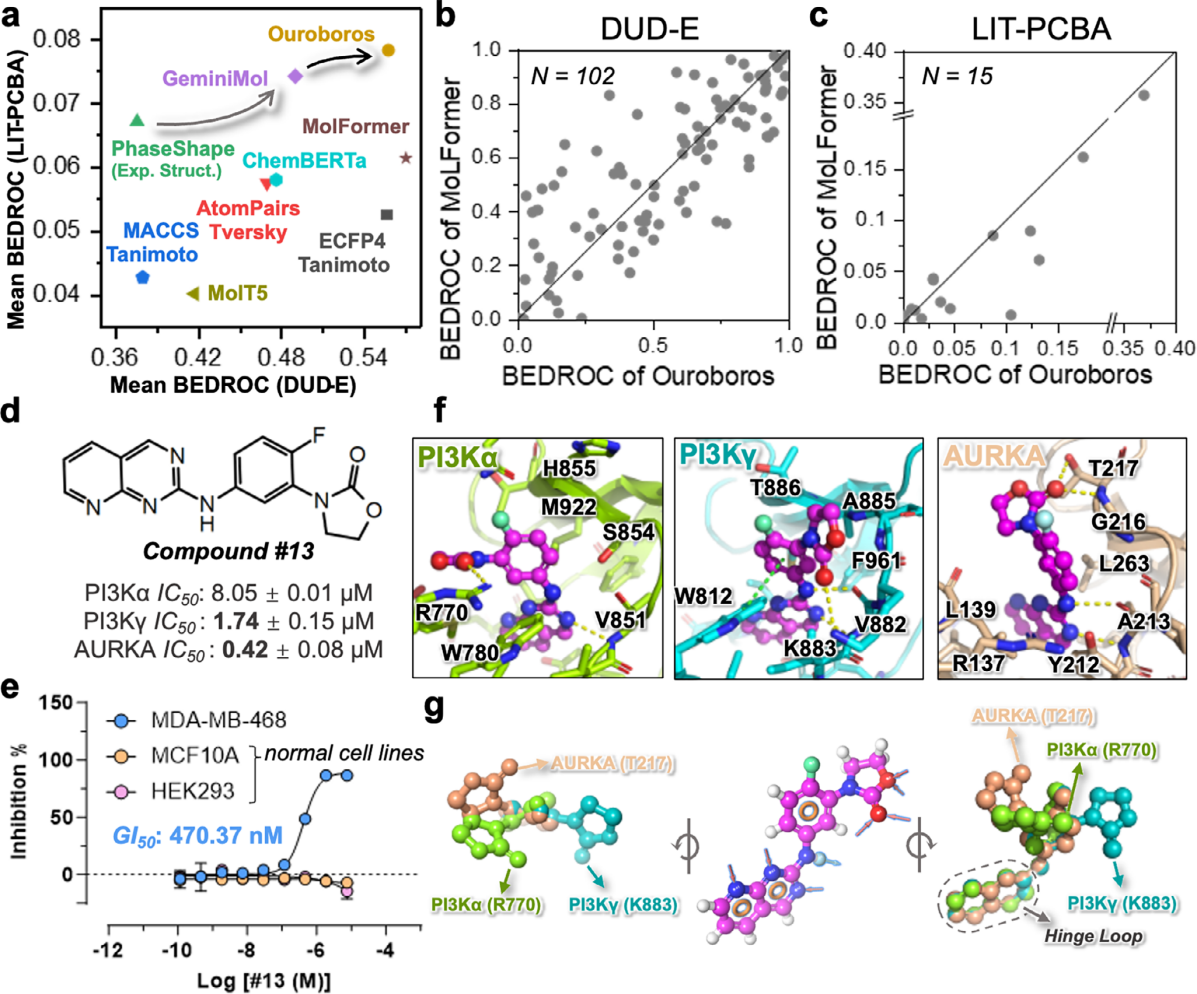
Applying the Ouroboros representation similarity to virtual screening and targeted poly‐pharmacology design. (a) Similarity‐based virtual screening performance evaluated by the mean BEDROC on the 102 targets from DUD‐E versus that on the 15 targets from LIT‐PCBA by different methods, including Ouroboros, GeminiMol, PhaseShape (conformer‐dependent), ChemBERTa (the best performance version of ChemMLM), AtomPairs, ECFP4, and MACCS. (b) Head‐to‐head comparison between Ouroboros with MolFormer on the 102 targets of DUD‐E benchmark datasets. (c) Head‐to‐head comparison between Ouroboros with MolFormer on the 15 targets of LIT‐PCBA benchmark datasets. (d) *IC*_50_ of hit compound #13 for three targets, including PI3Kα, PI3Kγ and AURKA. The *IC_50_* value is represented as mean ± SD. Two technical replicates were performed for each concentration. (e) Growth inhibition curve and *GI*_50_ of compound #13 in breast cancer (MDA‐MB‐468) and normal cell lines (MCF10A and HEK293). The *GI*_50_ value is represented as mean ± SD. Two technical replicates were performed for each concentration. (f) The compound #13 exhibits three different binding poses within the three target protein pockets. The 2‐oxazolidinone group form hydrogen bonds with different residues. (g) The analysis of superimposed conformer reveals relationships between 2‐oxazolidinone flipping and poly‐pharmacology of compound #13. The atom involved in hydrogen bonds are indicated by arrows.

Ouroboros demonstrated the representational capabilities over 6 baseline methods (Figure 2a) on an early enrichment metric called Boltzmann‐enhanced discrimination of receiver operating characteristic (BEDROC) [47] across two virtual screening benchmarks (102 targets in DUD‐E and 15 targets in LIT‐PCBA). The results show that despite the relatively small training structural dataset, our representation module achieves the balanced high enrichment scores in both benchmarks. Furthermore, as shown in Table S2, Ouroboros outperformed or showed comparable performance with the baseline methods on most of other metrics, including AUPRC, AUROC, enrichment factor (EF), and log AUC. Among all molecular base baselines, MolFormer [48] showed the best performance, and the ChemBERTa‐2 [23, 24] and MolT5 [49] are comparable with the molecular fingerprint.

As the molecular foundational model with the most massive pre‐training datasets (both ZINC and PubChem), MolFormer has demonstrated competitive performance in both DUD‐E and LIT‐PCBA. Therefore, we further compared Ouroboros with MolFormer [48] head‐to‐head on the DUD‐E and LIT‐PCBA, as shown in Figure 2b,c. Ouroboros was comparable to MolFormer in the DUD‐E benchmark and exhibited complementary performance on different targets, while in LIT‐PCBA derived from high‐throughput experimental screening, Ouroboros surpassed MolFormer on most targets. It is worth noting that Ouroboros only performs pre training on a 120 K molecular dataset, which requires it to learn principle that can generalize to a wide range of chemical spaces on a small number of molecular structures. This is fundamentally different from large‐scale pre training strategies such as MolFormer.

Benefiting from the introduced conformational spatial similarity, GeminiMol [19] and Ouroboros can outperform molecular fingerprints and experimental structure‐based PhaseShape [50] in the LIT‐PCBA benchmark. These results suggest that the Ouroboros representation are more effective on clustering molecules with similar pharmacological features in proximity within the representation space compared to the baseline methods. Such results further demonstrate that our representation module is sufficiently robust to be applied as a stand‐alone tool for small molecular similarity screening.

#### 2.3.3 Designing Poly‐Pharmacology Cancer Inhibitors

Given the learned conformational space information in representation module and Ouroboros’ outstanding performance in the DUD‐E and LIT‐PCBA benchmarks, we apply our model to a challenging poly‐pharmacology (also as multi‐target) drug discovery scenario to evaluate its practical utility. Noting that terminal cancers are often difficult to treat because of their heterogeneity, thus we focus on identifying poly‐pharmacology inhibitors for multiple cancer driver genes [51], such as KRAS, TP53, PI3Kα, SMAD4, and ARID1A, targeting synthetic lethality or promoting cell proliferation. Among them, PI3Kα and PI3Kγ are proto‐oncogenes, and they also play key roles in the downstream proliferative signaling pathway of KRAS [52]. AURKA is a synthetic lethal gene associated with oncogenes such as SMARCA4, ARID1A, RB1 [53], and SMAD4 [54], and mutations in these oncogenes play the role of drivers in a wide range of cancers [51].

We anticipated that Ouroboros would identify structurally novel inhibitors from the Enamine REAL diversity set [55], capable of simultaneously targeting at least 2 of these cancer drivers. A total of ten distinct driver‐related targets (KRAS, PI3Kα and PI3Kγ, MEK1/2, PLK1, WEE1, CHK1/2, AURKA, PRMT5, PARP1/2) and 119 reference compounds are included in this experiment. We first enrich potential active molecules through similarity screening, followed by molecular docking to select candidate compounds for experimental assay. It is notable that no candidate shares similarity with the reference compounds from all targets, and they usually will only show similarity with 2–3 targets. Among these targets, kinases are most likely to find candidates for poly‐pharmacology.

#### 2.3.4 Experimentally Identify the Potential Lead Compound #13

A total of 18 molecules are successfully synthesized for enzyme activity assay, of which seven compounds (38%) are active, and three compounds (16%) exhibited multi‐target inhibitory behavior as expected (Table S3). This means that this hit rate screening exceeded the median hit rate of 13.3% in the virtual screening [56]. Figure S8 summarizes the results of the three identified hit molecules, each achieving a maximum similarity of over 0.5 with the active reference molecules of three targets of interest, including PI3Kα, PI3Kγ, and AURKA, which demonstrates appreciable inhibitory activity in enzymatic assays.

Notably, compound #13 exhibited superior activity across all three targets, with an *IC*_50_ in the nanomolar range for AURKA, highlighting its potential as a promising lead compound. However, the testing results of all 18 candidate compounds (Table S3) indicated that none showed an ability to inhibit more than four targets simultaneously. This limitation may be attributed to the relatively low throughput of the experiment and the use of only a 48.2 m subset of the REAL library [55] during the screening. Additionally, the findings confirmed that compounds #2, #6, and #13 are not pan‐kinase inhibitors, which selectively inhibit PI3K and AURKA. Aiming to further validate the activity and safety of #13, we investigated its growth‐inhibitory activity in breast cancer cell line MDA‐MD‐468, mammary gland cell line MCF10A, and embryonic kidney cell line HEK293. As shown in Figure 2e, #13 achieved a GI50 of 470.37 nm against cancer cells while exhibiting no inhibitory effect on normal cells. Overall, we identified three multi‐target hit molecules from a 48.2 m compound dataset by Ouroboros combined with molecular docking. These results show the application potential of Ouroboros representation in similarity screening and further corroborate the ability of representation module to generalize to ultra‐large molecular structure datasets. This provides credibility to preform directed chemical evolution in Ouroboros’ molecular representation space.

#### 2.3.5 Retrospective Analysis

We remain curious as to how Ouroboros discovered compound #13 and whether different targets select for multiple conformations in its conformational space. This would reveal the significance of introducing conformational space information in Ouroboros. Figure 2f illustrates the binding mode of #13 in 3 targets, where 1,3,8‐ triazanaphthalene forms a hydrogen bond with the hinge loop region in all targets, while 2‐oxazolidinone undergoes a flip to form hydrogen bonds with different residues in the different targets, i.e., T217 in AURKA, R770 in PI3Kα, K883 in PI3Kγ. Inspection of aligned ligand conformations (Figure 2g) reveal that 2‐oxazolidinone undergoes a large flipping in all 3 conformations to support its hydrogen bond with side chain of different residues. It demonstrated the unique advantages of conformation‐independent AI model that learned conformational space information over conformation‐dependent approach in poly‐pharmacology.

### 2.4 Modeling Chemical Properties based on the Representation Module

#### 2.4.1 Molecular Property Modeling via Projection

**FIGURE 3:**
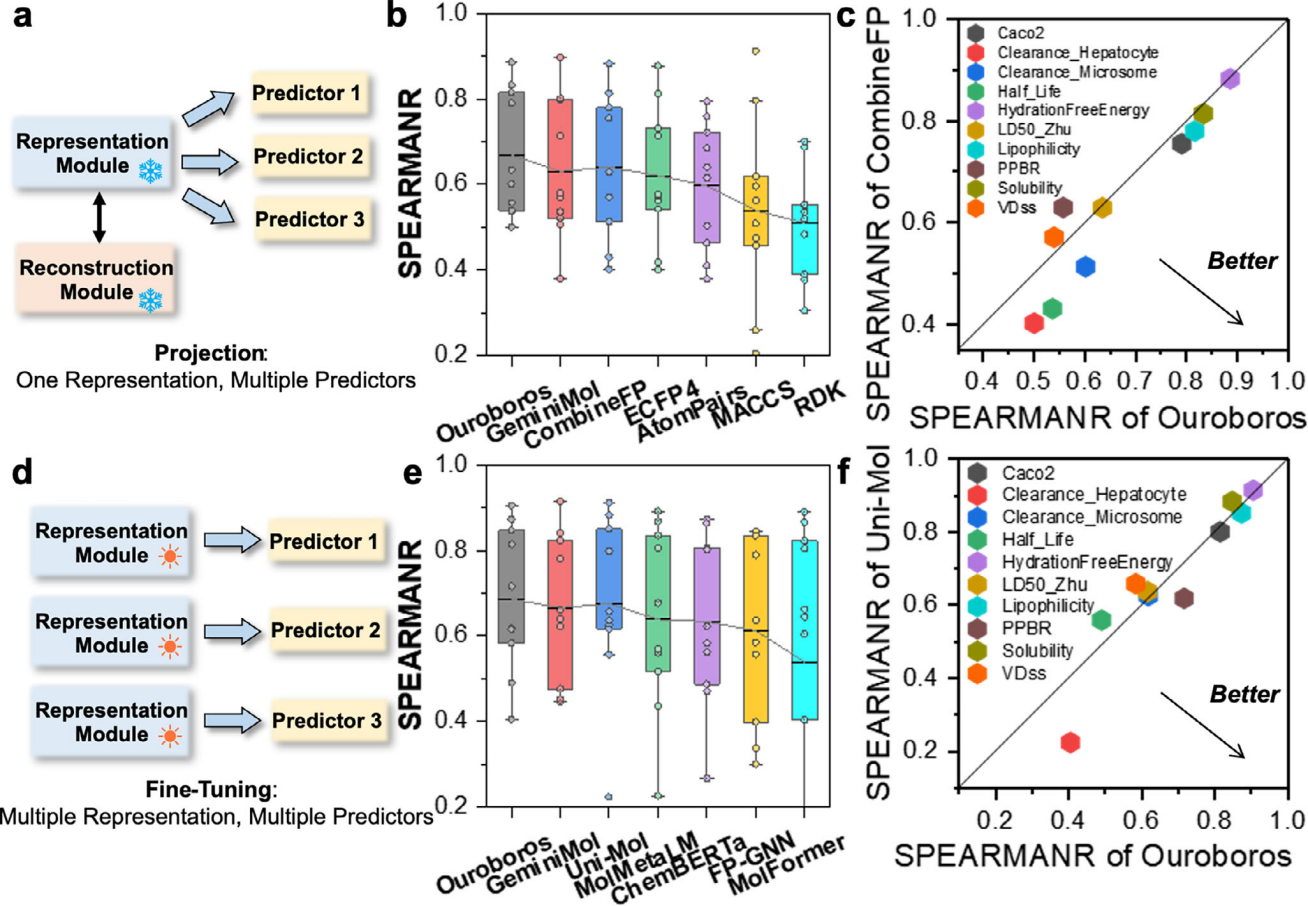
Training molecular property predictors based on Ouroboros representation module. (a) Projection strategy of molecular property predictor. (b) Spearman correlation coefficients achieved by 7 molecular representation methods on ten different molecular property regression tasks. For GeminiMol and Ouroboros, the property predictors were constructed using a multilayer perceptron. CombineFP combines four molecular fingerprints together, including AtomPairs, TopologicalTorsion, ECFP4, and FCFP6, which uses neural network to construct property predictors. (c) Spearman correlation coefficients by Ouroboros on ten different molecular property regression tasks (colored differently) versus that by CombineFP. (d) Fine‐tuning strategy of molecular property predictor. (e) Comparison of SpearmanR between Ouroboros with GeminiMol, geometric pre‐training baseline (Uni‐Mol), three chemical language baselines (MolMetaLM, ChemBERTa, and MolFormer), and QSAR baseline (FP‐GNN). (f) Head‐to‐head comparsion between Ouroboros with Uni‐Mol.

In Ouroboros, predicted chemical properties serve as the loss function guiding optimization. The regression‐based property predictor provides a continuous gradient of property strength, enabling more effective guidance for directed molecular migration. Here, the molecular property predictor in Ouroboros is implemented by mapping molecular representations to properties via deep neural networks (Figure 3a). Accordingly, in this work, we compared the performance of Ouroboros against baseline methods across 10 datasets of molecular properties related to pharmacokinetics and toxicology and examined the predictive ability of different models on the test set with the same data partition. Figure 3b presents the average Spearman correlation coefficient (SPEARMANR) of the Ouroboros predictor on ten different molecular property datasets, which is significantly higher than that of AI‐based GeminiMol [19] and fingerprint‐based methods, including ECFP4 [57], AtomPairs [58], MACCS [59], RDK [60], and CombineFP [19]. Meanwhile, Ouroboros achieved the highest SPEARMANR in 5 out of 10 property tasks among all baseline methods (Table S4). By projecting multiple property predictors, Ouroboros is empowered to perform backward optimization of molecular structures across multiple distinct molecular properties.

#### 2.4.2 Head‐To‐Head Comparison with CombineFP

Combined molecular fingerprints are one of the most powerful methods available for modeling molecular properties, and many recent studies have demonstrated that molecular fingerprints, as a traditional method, when coupled with effective machine learning methods can yield powerful generalization and prediction capabilities [61, 62]. As an illustration, Figure 3c shows a head‐to‐head comparison of Ouroboros with CombineFP, where Ouroboros not only excels in relatively simple tasks, such as lipophilicity and solubility prediction, but also achieves substantial improvements in more challenging tasks like half‐life and clearance rate. These results underscore the strong generalizability of Ouroboros representations, which are crucial for training effective property predictors across a diverse range of downstream molecular property tasks.

#### 2.4.3 Molecular Property Modeling via Fine‐Tuning

Fine‐tuning pretrained models on independent downstream tasks is a common benchmarking strategy, although this approach sacrifices Ouroboros' unique advantages in molecular generation. During fine‐tuning, each property predictor independently optimizes the representation module, rendering the pretrained reconstruction module no longer available (Figure 3d). To compare Ouroboros with other chemical language baselines, we evaluated its performance in fine‐tuning mode, as shown in Figure 3e and Table S5. Despite not being designed for fine‐tuning, Ouroboros still achieved the highest average performance (0.687) among all methods, followed by Uni‐Mol [18] (0.676). It also outperformed FP‐GNN [63], which was specifically designed for molecular property modeling by integrating multiple fingerprints with GNNs. We conducted a head‐to‐head comparison between Ouroboros and Uni‐Mol (Figure S3f). Both methods demonstrated distinct advantages across different tasks, with Ouroboros exhibiting superior clearance and plasma protein binding rate.

#### 2.4.4 Head‐To‐Head Comparison with GeminiMol on the Cellular Response Modeling

Given the superior performance of GeminiMol in modeling drug‐induced cellular responses, we compared Ouroboros and GeminiMol in predicting drug response, specifically cell growth inhibition *GI*_50_, across 60 cell lines from the NCI/DTP. A total of 56 326 compounds were collected and divided into structurally non‐overlapping training, validation, and test sets based on compound scaffolds, which were defined through clustering using molecular skeletons and fingerprints. The structures of most compounds are largely independent of those in the pretraining molecular datasets of both Ouroboros and GeminiMol (Figure S9a). Under the projection setting, the predictor was trained to simultaneously estimate the drug responses of all 60 cell lines. Ouroboros achieved Spearman correlation coefficients exceeding 0.6 across most tasks, and mean value is 0.657, versus mean value of GeminiMol is 0.607 (Figure S9b). We conducted a head‐to‐head comparison of them, with the results shown in Figure S9c. Ouroboros demonstrates improvements across all targets.

### 2.5 Directed Chemical Evolution in Representation Space for Molecular Generation

#### 2.5.1 Chemical Structure Propagation

To evaluate the effectiveness of directed chemical evolution, we first examine whether stochastic propagation can progressively modify the structure of the starting molecule and generate novel chemical scaffolds (Figure S10a). Using aspirin as an illustrative example, Figure S10b demonstrates that the molecular structures generated through this process gradually diverge from the initial molecule while maintaining an observable similarity. Meanwhile, their representation similarity decreases as propagation progresses. This finding suggests that exploring the neighboring regions of Ouroboros in the representation space can uncover structural analogs of the starting molecule, supporting the use of Ouroboros for molecular generation.

#### 2.5.2 Directed Chemical Evolution

The opposite of stochastic propagation is directed evolution, a strategy that employs a loss function to guide the directed optimization of representation vector, thus permitting the directed optimization of chemical structures (Figure S10c). As a demo, we examine directed molecular optimization guided by two specific properties: water solubility and membrane permeability. As shown in Figure S10d, starting from a hydrophobic molecule, Ouroboros successfully generates molecules with improved solubility while preserving overall structural similarity. Similarly, beginning with a highly flexible and strongly hydrophilic (led to poorly permeability) molecule, Ouroboros produced structures with reduced flexibility and carrying positive charges, which exhibited enhanced predicted membrane permeability. This result demonstrates that Ouroboros is capable of selectively optimizing specific properties of the molecules during the evolution.

#### 2.5.3 Directed Migration Explores Transformational Pathways of Chemical Structures

**FIGURE 4:**
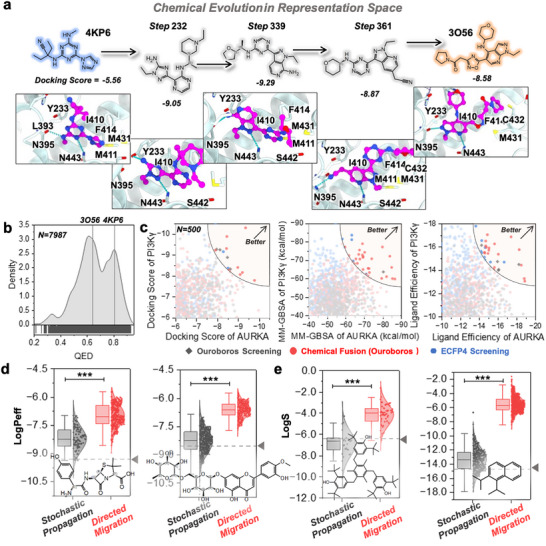
Exploring representation space within Ouroboros. (a) Chemical migration from one inhibitor of PDE4B to another. Docking scores are computed using Glide SP. The C atoms of the ligands are colored in magenta. (b) Distribution of QED scores for molecules generated during directed migration. The QED scores for the start and target molecules are displayed as vertical reference lines. (c) The chemical fusions in representation space for dual‐target drug discovery. Comparison of similarity‐based virtual screening (Ouroboros in grey and ECFP4 in blue) and chemical evolution (colored in red). Assessment of molecules generated using chemical fusion and similarity screening. The black diamonds represent molecules produced by Ouroboros similarity screening, the blue hexagons represent molecules produced by ECFP4 similarity screening and the red circles represent molecules produced by chemical fusion. The translucent sectors in the upper right corner represent high‐quality candidate molecules. (d, e) The comparison of stochastic propagation and directed migration in multi‐objective molecular properties optimization for membrane permeability (d) and solubility (e). All the points in the box plots represent new molecules with representation similarity greater than 0.6 generated during the propagation process and migration pathway. The predicted membrane permeability and solubility were evaluated by a third‐party program QikProp. The dashed line represents the starting molecule. “***” refers to *p*‐value < 0.0001.

Benefiting from the representation space established in representation learning, we can explore the chemical structures that lie between two compounds in terms of pharmacophore characterization for drug scaffold hopping. In Figure 4a, Ouroboros is utilized to map a migration pathway between the representation vectors of two inhibitors with distinct scaffolds ([1,3,5]‐triazine derivative and 5‐heterocycle pyrazolo pyridine), both targeting 3′,5′‐cyclic‐AMP phosphodiesterase 4B (PDE4B), a crucial regulator of various physiological processes [64]. Docking the molecular decoys generated along this pathway into the PDE4B binding pocket revealed that the intermediates retained similar binding poses to the two reference molecules. Notably, three intermediate molecules along the migration trajectory exhibited superior docking scores compared to both reference compounds, highlighting Ouroboros’ potential in scaffold hopping and structural optimization.

Since Ouroboros aims to train a reconstruction module faithful to the chemical space, it should indeed generate molecules with similar QED scores to the reference molecules during the migration pathway. We compared the QED scores of all compounds generated by chemical migration with those of two starting compounds. As shown in Figure 4b, Ouroboros produced a number of new molecules with QED scores exceeding those of the two reference molecules (1284 molecules, 16.08% of total). Besides, we compared the SA scores of molecules on the migration pathway with the reference molecule (Figure S11). Since we did not employ any predictors on the migration pathway to guide the model toward generating molecules with higher SA scores, we could not expect Ouroboros to produce candidates with high SA scores. However, thanks to the reconstruction module's effective modeling of chemical space, most molecules on the migration pathway achieved SA scores exceeding 0.5, which is generally considered synthesizable.

#### 2.5.4 Chemical Fusion Helps Design Dual‐Target Molecules

We have proved AURKA and PI3Kγ are kinase targets amendable for simultaneous inhibition. This finding motivated us to explore whether Ouroboros could integrate pharmacophore features from two sets of reference molecules to generate novel dual‐target inhibitors. To investigate this, we conducted similarity screening on the Enamine REAL diversity set [55] (the training dataset for reconstruction module) to identify potential dual‐target inhibitors for AURKA and PI3Kγ. In parallel, the same reference compounds were input into Ouroboros for chemical fusion, enabling a direct comparison between the candidate compounds generated by both approaches.

To address this, we retained partially fused molecules from the chemical fusion process that achieved a maximum similarity exceeding 0.65 for both targets. Among these, the top 500 molecules (ranked by synthetic accessibility) were selected and compared directly with the top 500 molecules from similarity screening via Ouroboros and ECFP4 in a head‐to‐head evaluation. To further assess the development potential of these candidate compounds, we performed molecular docking to evaluate their binding affinity and calculated MM‐GBSA (Molecular Mechanics Generalized Born Surface Area) binding free energy (Figure 4c). The results demonstrated that chemical fusion produced more compounds with superior docking scores and binding free energies than both ECFP4 and similarity screening. Additionally, we compared the ligand efficiency between the generated molecules and screened molecules via ECFP4 and Ouroboros (removing the effect of ligand size), further confirming the advantage of Ouroboros’ chemical fusion in identifying highly efficient dual‐target inhibitors. Overall, chemical fusion has the superior capability of lead compound screening over similarity screening, while this capability is expected to further improve as scaling up of the training dataset for reconstruction module.

#### 2.5.5 Drug Lead Optimization through Directed Migration

Benefitting from Ouroboros' orthogonal architecture, all property predictors can be directly employed for directed molecular optimization. Here, we compare the performance of Ouroboros stochastic propagation and directed chemical evolution in molecular property optimization using a third‐party evaluation method QikProp (Schrödinger, LLC). Figure 4d,e present solubility and membrane permeability optimization results on a broader scale, featuring four representative organic molecules: two with low membrane permeability (*Amoxicillin*, an antibiotic, and *Diosmin*, a natural product) and two hydrophobic molecules with poor water solubility (initial structures label on figure). The loss function was designed to simultaneously enhance solubility, membrane permeability, and lipophilicity, while maintaining structural similarity to the original molecules (as described in ***Methods***). Despite applying the same loss function to both categories of molecules with suboptimal properties, Ouroboros’ directed migration successfully generated multiple molecules with notable improvements in membrane permeability or solubility, while preserving an representation similarity above 0.6. These findings underscore the versatility and effectiveness of Ouroboros’ directed migration approach in molecular property optimization.

## 3 Discussion

We have developed a molecular foundational model, Ouroboros, with orthogonal architecture. The orthogonal architecture aims to assign the most suitable training strategies and model architecture to three independent modules, i.e., representation of chemical molecules, reconstruction of molecular structures from representation vectors, and chemical‐property relationships on specific biological problems. Thus, the representation module can utilize GNNs and similarity learning, the reconstruction module can employ an autoregressive Transformer‐based chemical language model. From there, Ouroboros can train property predictors related to practical applications, which can be employed to design loss functions and guide gradient‐based optimization of representation vectors and ultimately reconstruct the optimized molecular structure. Benefiting from the orthogonal architecture, the property predictors can adopt a suitable model for each task. Here, we uniformly employ deep neural networks in benchmark test, but more elaborate model architectures could be adopted in the future by incorporating other information, such as target embedding.

The representation module incorporates a similarity learning strategy that significantly enhances data efficiency, enabling effective pre‐training on a compact chemical dataset (<150 000 molecules) while demonstrating exceptional virtual screening performance on large‐scale benchmarks such as DUD‐E and LIT‐PCBA (>1 million molecules). Its real‐world utility was further validated through poly‐pharmacology drug discovery, successfully identifying three novel poly‐pharmacology inhibitors from a 48.2 million‐compound diversity library, showcasing its strong generalization across chemical spaces. Among them, compound #13 exhibits potential as a poly‐pharmacology lead compound, which despite its low molecular weight, interacts with different target residues through conformational changes, thus achieving the expected poly‐pharmacology effects.

The reconstruction module employs an auto‐regressive Transformer decoder [39] that reconstructs SMILES sequences from 1D representation vectors, enabling two generative strategies: stochastic propagation and directed chemical evolution. Benchmark evaluations on molecular property optimization and dual‐target inhibitor design confirm its superior performance. In this context, Ouroboros effectively bridges the gap between molecular representation learning and generative AI, establishing a new paradigm for continuous chemical evolution in representation space. Beyond these strategies, Ouroboros can also integrate other AI‐driven models, leveraging them as loss functions to optimize molecular properties.

Despite the success, many challenges remain in this framework. Ouroboros does not directly predict dynamic 3D conformations of molecules, which is a promising direction in the future. This design choice reflects the fact that the conformations of small molecules are inherently dynamic and diverse, owing to variations in rotatable bonds and environmental conditions. We recognize that predicting the dynamic conformational space of molecules represents an important and promising direction for future work. Second, while Ouroboros has shown superior performance in predicting multiple molecular properties, the current benchmark includes only ten molecular properties, indicating significant opportunities for future development. Moreover, there is increasing interest in generating molecules with strong binding affinity for specific biological targets. Currently, Ouroboros does not predict drug‐target binding affinity directly. Instead, it relies on molecular docking to identify molecules along the transition path with superior docking scores and binding poses. This highlights the keen need to incorporate protein representations into the training of drug‐target binding affinity prediction models to further enhance the capabilities of the Ouroboros framework.

## 4 Methods

The Ouroboros designs as unified molecular foundational model and employs three independent modules: 1) a representation module that represented molecular structures into 1D vectors (Figure 1a); 2) a reconstruction module that reconstructs molecular structures from 1D vectors (Figure 1c); and 3) property predictors that translate 1D vectors into molecular properties (Figure 1d). The protocol is flexible and can be easily extended to integrating other representation learning models with domain‐specific knowledge for molecular representation.

### 4.1 Model Architecture

#### 4.1.1 Representation and Massage Passing of Molecular Graph

To represent the chemical space, a representation module of 1D molecular representation with a size of 2048 is pre‐trained by molecular similarity learning, which promotes the model to identify molecules with similar chemical structures and pharmacophore features. For this, we first convert SMILES representations into molecular graphs, which are then processed by a GNN‐based Weisfeiler–Lehman Network (WLN) [65] for feature updating (Figure S1a). Next, a global self‐attention pooling module is applied to compute atom‐wise attention weights. These weights are finally used to aggregate atomic features into a molecule‐level representation vector, which can be further utilized for various pre‐training tasks, including fingerprint similarity and CSPS.

The small molecular structure in encoder is represented as a graph *G* = (*V*, *E*), with *V* representing atoms and *E* chemical bonds. The atom features include atom type, hybridization, formal charge, chiral tag, whether the atom is in a ring, and whether the atom is aromatic. The bond features include whether or not they are conjugated, ring‐forming, and chiral types. Both the construction of molecular graphs and the message passing are implemented through the DGL [66, 67] package.

As shown in Figure S1a, the GNN processes molecular graphs by iteratively updating atom and bond features through a message‐passing scheme. The input atom features $h_{v}^{\left(0\right)}$ are projected into a higher‐dimensional space using a linear transformation followed by a ReLU activation:

(1) $$h_{v}^{\left(0\right)}=ReLU\left(W_{\mathrm{in}}h_{\nu }+b_{\mathrm{in}}\right)$$

where *W*_**in**_ is the learnable weight matrix, and *b*_**in**_ is the bias term. For each bond feature *e*_uv_ contacting atoms *u* and *v*, a message is generated by concatenating the source atom features *h*_u_ and *e*_uv_:

(2) $$m_{\mathrm{uv}}=ReLU\left(W_{\mathrm{contact}}\left[h_{u}∥e_{\mathrm{uv}}\right]+b_{\mathrm{contact}}\right)$$

where ∥ denotes concatenation. These messages are used to update the bonds features:

(3) $$e_{\mathrm{new}}^{\mathrm{uv}}=m_{\mathrm{uv}}$$

The atom features are updated by aggregating messages from neighboring bonds and combining them with the atom's previous features:

(4) $$h_{v}^{\left(t+1\right)}=ReLU\left(W_{\mathrm{aggregate}}\left[h_{v}^{\left(t\right)}∥\sum_{u\in N\left(v\right)}e_{\mathrm{uv}}^{\mathrm{new}}\right]+b_{\mathrm{aggregate}}\right)$$

where N(v) is the set of neighbors of atom *v*, the *t* represents the times of message passing (four times in this work). Then, atom features *h*_v_ and bond features *e*_uv_ are independently projected into a latent space to prepare them for further interactions, element‐wise multiplicated, and aggregated from all neighbors of the atom *v*:

(5) $$h_{v}^{\mathrm{final}}=W_{\mathrm{self}}h_{v}⊙\left(\sum_{u\in N\left(v\right)}\left(W_{\mathrm{bond}}e_{\mathrm{uv}}⊙W_{\mathrm{atom}}h_{\mathrm{uv}}\right)\right)$$

The final atom representation combines the aggregated neighbor features and a self‐loop attention through element‐wise multiplication.

#### 4.1.2 Global Attention Pooling Module

The model implements global self‐attention pooling through the following steps: for each atomic features *h*_i_ in the molecular graph, a MLP predicts its attention weight, denoted as *a_i_*. These weights are normalized using the SoftMax function to ensure they sum to 1:

(6) $$w_{i}=\frac{\exp\left(a_{i}\right)}{\sum_{i}\exp\left(a_{i}\right)}$$

Each atomic feature is then scaled by its normalized attention weight, and the scaled features are summed to generate the graph‐level representation *H*_graph_:

(7) $$H_{\mathrm{graph}}=\sum_{\left\{i\in V\right\}}h_{i}w_{i}$$

As shown in Figure S1a, the MLP begins by projecting the atom features from 2048 to 6144 dimensions, followed by reductions to 1024 and 128 dimensions in sequence. The linear projection in 128 dimensions is repeated three times before projecting the features to a single dimension. Batch normalization is applied after the first two linear layers, and a sigmoid activation function is applied after every linear layer, including the final layer generating the 1D attention weight.

#### 4.1.3 The Projection Head of Representation Learning

The projection head of Ouroboros was designed to project the learned molecular representation into meaningful inter‐molecular similarities. The projection network consists of a series of fully connected layers with non‐linear activation functions and normalization (Figure S1b). Two representation vectors of query and reference molecules were input to the two projection heads of fingerprint similarity and CSPS. The representation vector first passes through a rectifier component, which expands the features to five times the dimension of the original representation vector, passes the LeakyReLU [68] activation function and a batch normalization layer. Subsequently, the expanded features are concatenated with the original representation vector and progressively passed through linear layers, sequentially reducing dimensions from 24 576 to 2048, followed by batch normalization. This process continues with a reduction from 2048 to 512 dimensions, another batch normalization step, and is repeated three times before being projected to the final output dimensions. The SiLU [69, 70] activation function was added between all linear layers in the projection head. Finally, it outputs a value that was reset to the range of 0–1 through a sigmoid neuron in the projection head.

#### 4.1.4 Reconstruction Module for Structural Reconstruction

The main role of the reconstruction module is to transform 1D representation vectors into probability distributions over tokens of a specific length. It is implemented by a Transformer decoder in PyTorch [71], and the 1D representation vector serves as the input memory. Using an autoregressive generation strategy, the decoder predicts the next token in the SMILES sequence until the end token occurred. We train a 4‐layer, 32‐head Transformer [39] decoder, equipped with layer normalization and SiLU [69, 70] activation functions, to reconstruct the SMILES by using the molecular representations as memory tensors. For the target SMILES, rotational position encoding and an embedding layer are utilized to generate target tensors, which are used for teaching‐force [39].

A deep neural network projects hidden states onto the token vocabulary during each prediction step (Figure S3). For the output features of the Transformer decoder, a 3‐layer MLP is employed to project these features to match the number of tokens. Specifically, the hidden features are first transformed to 4096 dimensions, subsequently reduced to 1024 dimensions, and finally mapped to logits over 43 distinct tokens.

#### 4.1.5 Representation of Chemical Language

The tokenizer used in reconstruction module is a Byte‐Pair [72] tokenizer, in which vocabularies were obtained by segmenting the SMILES of the compounds in our dataset. These tokenizers all include at least the basic elements that make up a pharmaceutical small molecule, such as C, N, O, S, P, c, n, o, s, F, Cl, Br, I, basic chemical bonds and isomer labels, such as, “=”, “@”, “/”, “∖”, and “#”, as well as other special symbols, such as brackets and numbers.

#### 4.1.6 Properties Predictor Module for Property Modeling

The property predictor in Ouroboros consists of three neural network components (Figure S12). The first component includes a dropout layer (dropout rate: 0.5) that projects the molecular representation from 2048 dimensions to 8192 dimensions, followed by a sigmoid activation function. The second component concatenates the molecular representation generated by the first component with the original molecular representation, resulting in a 10 240‐dimensional vector. This vector is processed through two linear layers while maintaining the feature dimension at 10 240, with LeakyReLU [68] activation and batch normalization applied between the layers. The third component performs an element‐wise addition of the input and output features from the second component. The resulting features are processed through a linear layer, batch normalization, and SiLU [69, 70] activation, then projected to 1024 dimensions. This is followed by another round of batch normalization and SiLU [69, 70] activation. Finally, the features pass through one more linear layer with SiLU [69, 70] activation to project them to the dimensionality of the target property (1D for all properties in the benchmark).

### 4.2 Data Collection and Training Scheme

#### 4.2.1 Molecular Datasets

We employed two molecular datasets of differing sizes to train and test representation and reconstruction modules for molecular chemical space representation. The first dataset contains 1 26 248 distinct molecules, collected from five categories of resources: 1) The diverse molecular dataset previously utilized in GeminiMol [19] which includes molecules from the Protein Data Bank (PDB) [73], GPCR Ligand [74], DEKOIS2 [75], chemical diversity set from Enamine, Glide Decoys from Schrödinger and Macrocyclic compounds from ChemBridge; 2) molecular dataset in the Human Metabolome Database [76]; 3) A macrocyclic peptide dataset CycPeptMPDB [77]; 4) The diverse molecular dataset in the Cell Painting Gallery [78, 79]; 5) A manual‐collected dataset of common biological cofactors, polypeptides, and oligonucleotides. Out of the 126 248 query molecules, 4728, 32, and 64 reference molecules are randomly selected and paired with the query molecules to form three similarity matrices of 4728  ×  126 152, 32  ×  512, and 64  ×  512, which are used as training, validation, and test datasets, respectively, for the representation module. Here, molecules in the validation and test sets were excluded not only along the reference‐molecule dimension (4728, 32, 64) but also along the query‐molecule dimension (126 248‐32‐64 = 126 152). When splitting the molecule datasets, we used a Tanimoto similarity cutoff of ECFP4 < 0.3 to ensure that the molecules in the validation and test sets are non‐homologous to the training set. For both the validation and test sets, we retained the 512 query molecules with the highest CSPS to enrich the most representative samples.

The second dataset is a combination of the 48.2 m Enamine REAL Diversity set [55] and the core dataset of the 126 248 molecules, which are designed for molecular reconstruction module development. Here, the validation set comprised 200 molecular structures from the REAL dataset, 256 structures from the first four subsets in core molecular dataset, and 35 molecules in the 5th subset, while all remaining molecular structures were used for training.

#### 4.2.2 Molecular Similarity Matrices

We designed two types of inter‐molecular similarity scores, CSPS and fingerprint similarity, for molecular similarity learning. All molecules in the core molecular dataset (126 248) are preprocessed, with calculations including the predictions of protonation under pH = 6.9 and tautomeric states by the LigPrep module [80] in the Schrödinger software package. For each molecule, the protonated and tautomeric state with the lowest penalty score was selected.

For a given pair of molecules, the CSPS is determined by identifying the conformations with the highest pharmacophore similarity when superimposing all conformations of one molecule onto those of the other. In our previous work on GeminiMol [19], we implemented a simplified version of CSPS descriptors, where conformational space exploration was constrained using two energy window scaling factors (0.5060 and 1.4806 kcal/mol). To generate pseudo‐labels for data augmentation, we preserved the minimum similarity during similarity calculation, which was used to estimate the conformational flexibility. While this pseudo‐labeling approach lacks a clear physical interpretation, the application of CSPS descriptors led to improved model performance, suggesting that a more refined CSPS descriptor could further enhance model accuracy.

In Ouroboros, we employ a more detailed conformational search and CSPS calculation approach by incorporating four energy windows, which are defined by multiplying the number of rotatable bonds with scaling factors of 0.14806, 0.5060, 0.88836, and 1.4806 kcal/mol. Among these four scaling factors, 0.5060 represents the first percentile of the ligand conformational strain energy in the PDB, 1.4806 denotes the mean, 0.14806 is 10% of mean (defined as the near‐lowest energy conformation), and 0.88836 is 60% of mean (defined as the medium‐energy conformation). Conformational searching is carried out using the Monte Carlo Multiple Minima (MCMM) [81] algorithm from the MacroModel module [82, 83] within the Schrödinger software package, executing 10 000 steps under the OPLS4 force field [84], with redundant conformations removed based on a 0.5 Å RMSD cutoff.

Superimposition and similarity calculations are performed using PhaseShape [50], where molecules are represented in pharmacophore of conformational space. PhaseShape is a well‐established tool for pharmacophore and molecular shape screening in drug discovery. We adopted it for Ouroboros modeling with specific aim of optimizing for drug discovery‐related applications, thereby minimizing potential bias conflicts between PhaseShape and the intended objectives of our model.

During data augmentation, numerical calculations were streamlined by retaining only the difference between CSPS values at the maximum and minimum energy windows. This strategy effectively captures molecular flexibility trends as a function of energy variations. Compared to GeminiMol [19], the enhanced conformational search with additional energy windows, lower RMSD thresholds, and an optimized search process, is expected to improve the CSPS calculation accuracy and physical interpretability without requiring complex data manipulations.

To construct the fingerprint similarity matrix, three descriptors (ECFP4 [57], MACCS [85], and AtomPairs [40]) are computed for each of the molecule pairs. For ECFP4 [57] and MACCS [85], we employed the Tanimoto similarity measure, while for AtomPairs [40], the Tversky similarity measure was chosen, as it demonstrated superior virtual screening performance in prior evaluations [19]. To augment the data, the difference in similarity under the largest and smallest energy windows was computed and added to the similarity vectors.

Consequently, the final similarity representation for each molecule pair contains five CSPS descriptors and three fingerprint similarities.

#### 4.2.3 Similarity Learning for Molecular Representation Pre‐Training

In the pre‐training task of similarity learning, we train the representation module by continuously sampling data from the similarity matrix (126 152 × 4728 in training set). In each batch, there are 512 query and 48 reference molecules, with eight different similarity labels for each pair of molecules. The model is thus trained to predict a third‐order matrix (512 × 48 × 8), with the loss function being MSE between predicted and actual similarity scores.

During the similarity learning stage, training is performed using the AdamW [86] optimizer in PyTorch [71], with a learning rate of 5.0 × 10^−^⁵ and a weight decay of 0.01. Two learning rate adjustment strategies are applied: 1) gradual increase at the beginning of training, and 2) cosine learning rate scheduling, which dynamically adjusts the learning rate based on training progress to prevent convergence to local optima. In the representation module, the WLN parameters are inherited from the GeminiMol model and remain frozen for the first 2000 training steps. The learning rate warmup starts at 10% of the original learning rate and increases linearly to the full learning rate over 10 000 steps. The minimum learning rate in the cosine learning rate schedule is set to 50% of the original learning rate, with a scheduling period of 10 000 steps. Additionally, an early stopping strategy is employed, where validation set performance is monitored every 200 steps. If no improvement is observed over 60 validation iterations, training is terminated early to prevent unnecessary computation.

#### 4.2.4 Molecular Property Modeling

The training, validation, and test sets for the molecular property predictor are divided according to molecular scaffolds, ensuring that approximately 20% of the molecules in the test set possess novel scaffolds not present in the training set. During the training stage, MSE loss function and AdamW [86] optimizer with a learning rate of 5.0 × 10^−5^ was used in training. The batch size is determined by the size of the training dataset: a batch size of 96 is used for training sets exceeding 5000 samples, 64 for those with more than 1000 samples but fewer than 5000, and 48 for training sets with fewer than 1000 samples. The learning rate scheduling strategy employed during training is identical to that used in pre‐training; specifically, the period of learning rate schedule is set to 2000 steps for training sets larger than 5000 and 1000 steps for smaller training sets. Model performance is validated every 200 steps, and training is early stop if no performance improvement is observed over 60 cumulative validation evaluations.

#### 4.2.5 Training Scheme for Reconstruction Module

In the training stage of the reconstruction module, causal masking is applied to implement teacher‐forcing [39]. To align its shape with the output tensor and integrate positional information, the teacher tensor is passed through an embedding layer followed by rotary positional encoding [87, 88]. The training process uses the previously described learning rate scheduling strategy, with a period of 4000 steps. The AdamW [86] optimizer is employed with a learning rate of 5.0 × 10^−5^ and a weight decay of 0.01.

The training loss for the reconstruction module is computed using a weighted cross‐entropy loss. The start‐of‐sequence token is excluded from the loss calculation. For each token *t*_i_, its weight is computed as inversely proportional to its frequency *f*_i_ in the batch relative to the total token count, capped at 100, i.e.,

(8) $$w_{i}=min\left(\frac{N_{\mathrm{total}}}{f_{i}},100\right)$$

where *N*_total_ is the total token count in the batch. If *f*_i_ = 0, *w*_i_ is reset to 1. Padding tokens, indexed as 0 in the vocabulary, are ignored during loss computation. The token‐wise cross‐entropy loss (excluding padding tokens) matrix $L_{\mathrm{CrossEntropy}}$ is multiplied elementwise by the weight matrix *W*:

(9) $$L_{\mathrm{tokens}}=L_{\mathrm{CrossEntropy}}⊙W$$

The final loss is obtained by averaging over all tokens (*j* is ground‐truth token):

(10) $$L_{\mathrm{final}}=\frac{\sum_{i,j}L_{\mathrm{tokens}}\left[i,j\right]}{\left|T_{\mathrm{vaild}}\right|}$$

where$\;|T_{\mathrm{vaild}}|$ is the number of valid tokens (excluding start‐of‐sequence token).

### 4.3 Downstream Tasks for Three Independent Modules

#### 4.3.1 Virtual Screening

In this study, we assessed the generalization capabilities of representation modules using the DUD‐E [45] and LIT‐PCBA [46] virtual screening benchmarks. In the benchmark tests of LIT‐PCBA [46], there are multiple active compounds for each target that can serve as reference molecules. Traditional baseline methods included ECFP4 [57], MACCS [85], AtomPairs [40], and Phase Shape [50], while GeminiMol served as the AI‐based baseline due to its use of a similar training strategy. For GeminiMol [19], the Pearson correlation coefficient, as described in its original study, was used as the similarity metric. In the case of Ouroboros, ChemBERTa, MolT5, MolFormer, and MolMetaLM [33], cosine similarity was applied due to its computational simplicity. Both Pearson and cosine similarity yield nearly identical performance for GeminiMol and Ouroboros, this is because the feature vectors produced by these models have mean values very close to zero at each dimension (Figure S6), resulting in highly similar numerical outputs between the two similarity measures. In this study, we use three different versions of ChemBERTa [23, 24], including the base model pre‐trained on 100K SMILES, and the two models Masked Language Modeling (MLM) and Multi‐Task Regression (MTR) pre‐trained on 77 M SMILES in ChemBERTa‐2 [23, 24], respectively. When using ChemBERTa to extract molecular coding, the batch size is set to 1 to avoid the effects of padding.

For each query molecule *q*, we calculate the similarity to all reference molecules *R* and select the highest similarity value as the final score, i.e.:

(11) $$sim\left(q\right)=\max_{\left\{r\in R\right\}}\left(sim\left(r,q\right)\right)$$

In this work, the BEDROC was used in the virtual screening as the evaluation metric, where the α of BEDROC was set to 160.9, corresponding to EF_1%_ [47].

#### 4.3.2 Chemical Visualization via t‐SNE

To test whether Ouroboros can separate molecules with different properties in the representation space, we performed feature dimensionality reduction and visualization on molecules in three chemical property classification datasets. First, we use Ouroboros' representation module to extract the representation vectors of all molecules, and analyze the standard deviations of all features. We extract the top 10% features with the largest standard deviation and project these features into 2 dimensions using t‐SNE. Finally, all molecules on the 2D plane were labeled with different colors based on their properties.

#### 4.3.3 Molecule Property Prediction (Projection)

To further evaluate molecular property modeling performance, we tested various models on ten diverse drug property datasets from TDC [89]. In this study, training, validation, and test sets were split based on molecular scaffolds, following the default data partitioning settings of TDC [89], with 20% of the data reserved for the test set. Both Ouroboros and GeminiMol [19] use frozen representation modules, where fixed molecular representations are directly projected onto molecular properties. CombineFP extracted 2048‐dimensional molecular fingerprints, including ECFP4 [57], FCFP6 [57], AtomPairs [40], and TopologicalTorsion [90], using RDKit [60]. These fingerprints are then transformed to property prediction via a deep neural network.

#### 4.3.4 Molecule Property Prediction (Fine‐Tuning)

We also compared the performance of Ouroboros with other baseline models in fine‐tuning mode. During the fine‐tuning, representation module (also called Encoder) of all models will be optimized. The ChemMLM, which performed well in similarity screening, were selected as representative models of ChemBERTa. In addition, MolFormer, Uni‐Mol, MolMetaLM [33], and FP‐GNN were also used as baseline model and tested with their default parameters and evaluated using the same data splits as the other approaches.

#### 4.3.5 Cellular Drug Response Prediction

We conducted a head‐to‐head comparison of GeminiMol and Ouroboros in their capability to model the *GI*_50_ property across 60 cell lines derived from the NCI/DTP dataset. This dataset originates from high‐throughput cell growth inhibition assays and contains a total of 56 326 diverse compounds. We extracted their skeletons and generated scaffold labels through clustering based on molecular fingerprints. Compounds with anyone identical labels were grouped into the same cluster, and the training, validation, and test sets were divided according to the cluster labels. The purpose of this procedure was to minimize the model's performance gains from memorizing chemical structures. Compared with the skeleton‐based splitting commonly used in benchmark tests, this approach is more advanced. In clustering, we used CombineFP and ECFP4 as molecular fingerprint extraction methods, and employed Euclidean distance and cosine distance for hierarchical clustering. The number of clusters was set to 10% of the total dataset size. We conducted three independent rounds of data splitting, training, and testing, and reported the average performance on the test sets.

#### 4.3.6 Molecular Structure Generation

For the reconstruction module, we reconstructed molecular structures from 1D vectors in the validation set and evaluated their validity and similarity to the original molecules. For this task, the molecular datasets selected were from first four subsets of the core molecular dataset. These experiments assessed whether the model could maintain molecular validity while producing structural diversity.

We examined the model's ability to generate diverse molecular structures by introducing 1) random Gaussian noise into 50% of the positions in the molecular representation vectors and 2) increasing the temperature of the autoregressive decoding process by Gumbel‐max [44] technique. This method incorporates stochasticity into the decoding process while respecting the probability distribution determined by the model.

For the first validation approach, we used Gaussian noise and scaled by $N\;\in \;\left[0.001,\;0.0025,\;0.005,\;0.0075\right]$. The perturbed representation vector *v* can be represented as:

(12) $$v=E+N\cdot \left(n⊙m\right)$$

where *n* ∼ **N**(− 1, 1), and for each position *i* of *E*, the mask *m*_i_ is defined as:

(13) $$m_{i}=\left\{\begin{array}{c} 1,with\;probability\;0.5\;\\ 0,otherwise\; \end{array}\right.$$

For the second approach, we used the temperatures T∈[0.01,0.1,0.5,1.0,1.5,2.0]. To sample token indices from a predicted probability distribution, we first apply the SoftMax function with a temperature *T* > 0 to smooth or sharpen the distribution. The temperature‐scaled log probabilities are computed as:

(14) $$\log p=\log SoftMax\left(\frac{y}{T}\right)$$

where *y* is the predicted logits, and *p* represents the normalized probabilities.

To enable sampling, we add Gumbel noise to the log probabilities. The Gumbel noise *g* is generated as:

(15) $$g=-\log\left(-\log\left(u+\in \right)\right)$$

where *u* ∼ Uniform(0, 1) and ∈ is a small positive constant to avoid numerical instability. The noisy log probabilities *s* are given by:

(16) s=logp+g

The sampled indices are obtained by taking the argmax over the *s*.

### 4.4 Applying the Ouroboros Representation in Practical Drug Discovery

#### 4.4.1 Biological Hypothesis and Reference Molecules

In this study, a total of 5 cancer drivers are selected for multi‐target drug screening. For KRAS, apart from itself, PI3Kα, PI3Kγ, MEK1/2, and PLK1 are selected as relevant targets. For TP53, synthetic lethal targets, WEE1 and CHK1/2, are selected. For SMAD4, AURKA is selected as a synthetic lethal target. For CDKN2A/MTAP, PRMT5 is selected as a synthetic lethal target. For BRCA1/2, PARP1/2 is selected as the synthetic lethal target. We retrieve all molecules from ChEMBL with pChEMBL values greater than 7.0 for the relevant targets. These compounds are then clustered using AtomPairs fingerprints and visually inspected to ensure structural diversity. Ultimately, 119 compounds are selected as reference molecules.

#### 4.4.2 Similarity Screening using Ouroboros Representation

In similarity screening, cosine similarity is used as the calculation metric, retaining only the maximum similarity for each reference molecule within a target set, as described in Equation 11. For query molecules with a similarity score below 0.5, the similarity value is reset to 0. Finally, the top 0.1% of molecules, ranked by the sum of similarities, are selected for subsequent molecular docking validation.

#### 4.4.3 Docking Validation

Although Ouroboros demonstrated superior early enrichment rates in benchmark tests, however, selection of a small number of optimal candidates by careful docking inspection is necessary due to the high cost of chemical synthesis. We further enriched potential active molecules using a hierarchical virtual screening process called GVSrun (https://github.com/Wang‐Lin‐boop/CADD‐Scripts/blob/main/GVSrun) in which the docking program is Glide [91]. The resulting docking poses were visually inspected to exclude irrational structures, which mainly include unsaturated hydrogen bond donors, ligand strain energies, and solvent effects.

#### 4.4.4 Enzyme Inhibitory Assay

A total of 18 compounds are successfully synthesized and subjected to enzymatic activity assays. The selected kinases for single‐dose enzymatic screening (10 µm) include PIK3CA, PIK3CG, AURKA, MEK1, CHK1, PLK1, and WEE1, while PIK3CA, PIK3CG, and AURKA are further assessed for IC_50_ determination. The enzymatic activity assay is conducted in a 384‐well plate (Greiner, 784075) at 25°C for 60 min. Each well contains 1 µm substrate, 1 nm kinase, and 4 µm ATP in assay buffer. After the reaction, 10 µL of kinase detection reagent is added to each well, followed by incubation at 25°C, centrifugation at 1000 rpm for 1 min, and a final incubation at 25°C for 1 h. Fluorescence signals are measured at 620 nm (Cryptate) and 665 nm (XL665) using a BMG plate reader.

#### 4.4.5 Cell Viability Assays

Cell proliferation was assessed using a luminescent cell viability assay. Briefly, cells were seeded in 384‐well plates and treated with compounds for 5 days under standard culture conditions (37°C, 5% CO_2_). Following the incubation period, the cell viability reagent was added, and the luminescent signal was measured using a microplate reader.

### 4.5 Implementation of Stochastic Propagation and Directed Chemical Evolution

#### 4.5.1 Stochastic Propagation

By incrementally introducing noise to the representation vector of the initial molecule, the progressive evolution of molecular structures can be observed. In the stochastic propagation experiment, we generate new molecular structures by adding random noise to the coding vector (Figure S7a).

For the entire representation vector *E*, we generate a standard Gaussian noise *n* ∼ **N**(− 1, 1), which was scaled by the standard deviation at each location, denoted as σ. The noise is further scaled to 0.05 and masked randomly at 50% of the locations. The perturbed representation vector *v* is represented as:

(17) $$v=E+0.05\cdot \left(n⊙\sigma ⊙m\right)$$

where the mask *m* is defined same as previous Equation 13.

The propagation undergoes a total of 200 steps, with the molecular structure at each step generated using a reconstruction module operating at a temperature of 0.30. As the number of steps increases, newly generated structures are incrementally accumulated, enabling thorough exploration of the local representation space.

#### 4.5.2 Directed Migration

Molecular representation vectors can be optimized using techniques similar to training neural networks, where the representation is directionally adjusted to optimize molecular properties as defined by the loss function. Starting from the initial molecular representation vector, the optimization is performed using the AdamW [86] optimizer with a learning rate of 2.0 × 10^−5^ for 600 steps. After each step, the representation vector is converted into a molecular structure using a reconstruction module with a temperature of 0.4 (Figure S7c).

The loss function consists of two terms. The first term minimizes the absolute difference between the *Sim*_start_ and a target value of 0.8, ensuring that structural changes are limited to preserve key pharmacophore features:

(18) $$L_{\mathrm{sim}}=|sim_{\mathrm{start}}-0.8|$$

where *Sim*_start_ is the molecular representation similarity between current representation with start molecule. The second term is defined by

(19) $$L_{\mathrm{prop}}=\sum_{\left\{j\in J\right\}}\left|c_{j}-f_{\mathrm{pro}p_{j}}\left(E\right)\right|$$

where *f*_prop_(*E*) represents the predicted molecular property, and *c_j_* is the predefined target value for property j∈J. This formulation guides the optimization process while maintaining a balance between multiple objectives. In this study, *J* consists of three molecular properties: solubility, membrane permeability, and lipophilicity.

The total loss function is expressed as:

(20) $$L=\frac{count\left(J\right)}{0.2}\times L_{\mathrm{sim}}+L_{\mathrm{prop}}$$

Besides optimizing molecular properties, directed migration can be used to migrate one molecular structure to another and to observe changes in molecular structure along the migration pathway within the representation space constructed through representation learning. This method is applicable to scaffold hopping, which involves transitioning between molecular scaffolds, and to the fusion of scaffolds with two distinct properties. The loss function can be expressed as:

(21) $$L=1-sim_{\mathrm{target}}$$

where *sim*_target_ is the similarity between current representation vector and the target molecular representation.

In the practice of scaffold hopping, the molecules generated from the directed migration are docked into the PDE4B protein structures (PDB code: 4KP6 [92] and 3O56 [93]) using the Glide SP (Standard Precision) [91] docking tool to evaluate their docking scores. The docking pose is visualized by ChimeraX [94].

#### 4.5.3 Chemical Fusion

Chemical fusion is an alternative approach to molecular generation by combining structural elements from reference compounds. Similar to directed migration, chemical fusion also uses optimizers and loss functions. Starting from the initial molecular representation vector (enumerated from reference molecules), the optimization is performed using the AdamW [86] optimizer with a learning rate 3.0 × 10^−5^ for 1000 steps. After each step, the representation vector is converted into a molecular structure using a reconstruction module with a temperature of 0.4 (Figure S7c).

For reference molecules r∈R and current molecular representation *E*, the similarity loss is denoted as:

(22) $$L=\min_{\left\{r\in R\right\}}\left(\frac{1}{1+10000^{sim\left(E_{r},\;\;E\right)-0.6}}\right)$$

where the *E*_**r**_ is the molecular representation of *r*. L could be considered as the minimum distance between the current molecule and the reference molecule, and minimizing this distance could produce a fusion between the molecules.

In the practice of multi‐target drug design, the molecules generated from the chemical fusion are docked into protein structures of AURKA (PDB ID: 5DT0 [95]), PI3Kα (PDB ID: 7MLK), and PI3Kγ (PDB ID: 3ML8 [96]) using the Glide SP (Standard Precision) [91]. Subsequently, the Prime module in the Schrödinger software package was used to calculate the MM‐GBSA binding free energy Δ*G*_MM − GBSA_ between the docking pose and target protein, where amino acids within 5 Å around the ligand are set as flexible regions and energy minimization is practiced. Finally, the ligand efficiency *LE* is calculated by:

(23) $$LE=\frac{\Delta G_{\mathrm{MM}-\mathrm{GBSA}}}{1+ln\left(N_{\mathrm{heavy}\;\mathrm{atoms}}\right)}$$

where *N*_heavy atoms_ refers to number of non‐hydrogen atoms. Here, LE is used solely as a virtual normalization metric to penalize excessively large molecules during model training and molecular generation, rather than to approximate experimental binding free energies. To reflect the nonlinear relationship between ligand size and potency commonly observed in medicinal chemistry [97, 98], we employ the natural logarithm of the heavy‐atom count as an empirical scaling factor.

#### 4.5.4 Statistical Analysis

For the data analysis of enzymatic and cellular assays, the percent inhibition for each compound was calculated relative to the DMSO (high control, 0% inhibition) and medium‐only (low control, 100% inhibition) controls. The assay quality was validated by calculating the Z'‐factor. Dose‐response curves were then generated by fitting the inhibition data to a four‐parameter logistic model (non‐linear regression). The *IC*_50_ and *GI*_50_ was determined from the fitted curves using a variable slope (four‐parameter model) in GraphPad Prism (v.8.0). The produced *IC*_50_ and *GI*_50_ values are represented as mean ± SD. For the data analysis of molecular properties optimization, two‐sample t‐test is used to assess significant differences in Origin 2021. The generated molecule number for stochastic propagation/directed migration are 111/390 for amoxycillin, 1239/610 for diosmin, 30/56 for diisopropyl naphthalene, 323/649 for ionox 330.

## Author Contributions

L.W. and Y.Z. conceived the project and designed the experiments; L.W. developed methods and designed and performed experiments; Y.W., H.L., M.L., Y.Z., C.C., C.L., and J.Z. helped with benchmark test, data collection, and insightful discussion; L.W. wrote the initial manuscript; Y.Z. revised the manuscript; all authors proofread and approved the final manuscript.

## Conflicts of Interest

H.L., M.L., C.C., C.L., and J.Z are affiliated with DeepMed Technology (Suzhou) Co., Ltd, but this affiliation did not influence the study design, data analysis, or interpretation of results. The authors declare no other competing interests.

## Supporting information




**Supporting File**: advs73595‐sup‐0001‐SuppMat.pdf.

## Data Availability

The data that support the findings of this study are openly available at (GitHub)[https://github.com/Wang‐Lin‐boop/Ouroboros] and (ZhangLab)[https://zhanggroup.org/Ouroboros/].
