# Supplementary material for: Learned Conformational Space and Pharmacophore Into Molecular Foundational Model
Source: Adv Sci (Weinh). 2026 Jan 4;13(17):e13556. doi: 10.1002/advs.202513556 (PMC13042461; doi:10.1002/advs.202513556)
Supplement: Supplementary file 1 — Supporting File: advs73595‐sup‐0001‐SuppMat.pdf. [file ADVS-13-e13556-s001.pdf]

Supplementary Information for

Learned Conformational Space and Pharmacophore into Molecular Foundational Model

Lin Wang, Yifan Wu, Hao Luo, Minglong Liang, Yihang Zhou, Cheng Chen, Chris Liu, Jun Zhang, Yang Zhang

This file includes:

Supplementary Figures S1 to S10
Supplementary Tables S1 to S5

Supplementary Figures

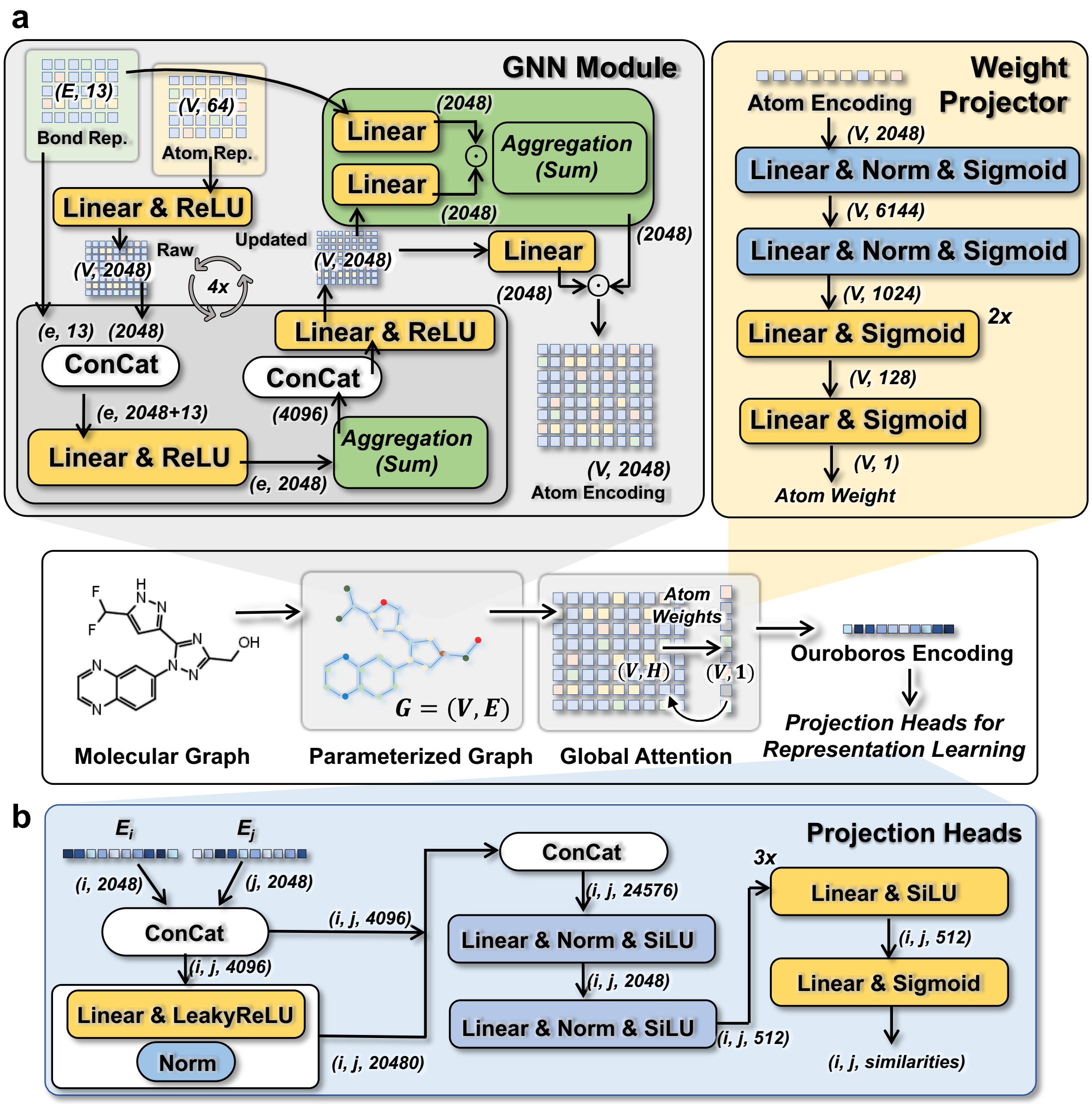

Figure S1 | The similarity learning strategies for representation learning of Ouroboros. (a) The overall architecture of the representation module. The representation of atoms includes one hot coding for atom type, hybridization, formal charge, chirality, and whether or not the atom is in a ring and aromatic. Chemical bond representations include whether or not they are conjugated, ring-forming, and chiral types. GNN module for massage passing of molecular graph. Weight projector for global attention module. Both GNN module and weight projector are used in representation module, the GNN components in the gray rounded rectangle are reused 4 times. The 'E' refers to bonds, 'V' to atoms, 'e' to bond of centor atom, 'B' to batch size. (**b**) The architecture of the projection head for inter-molecular similarities. The 'i' and 'j' refer to reference and query molecules.

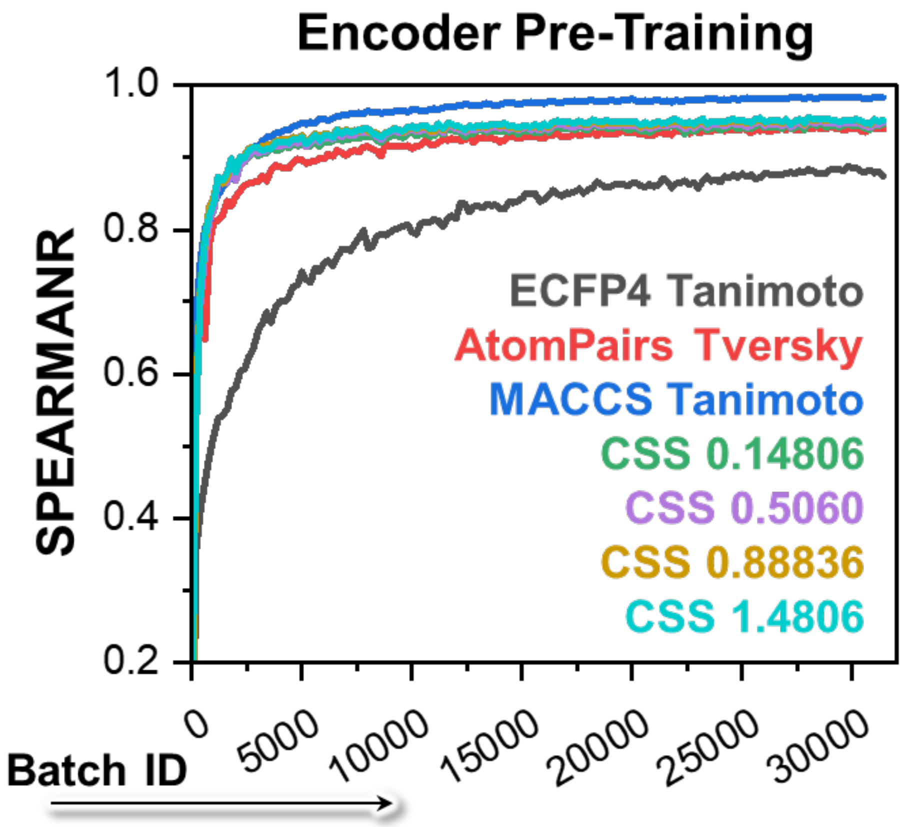

Figure S2 | The curve of Spearman's correlation coefficient variation for the validation set during training. The model was validated every 200 steps.

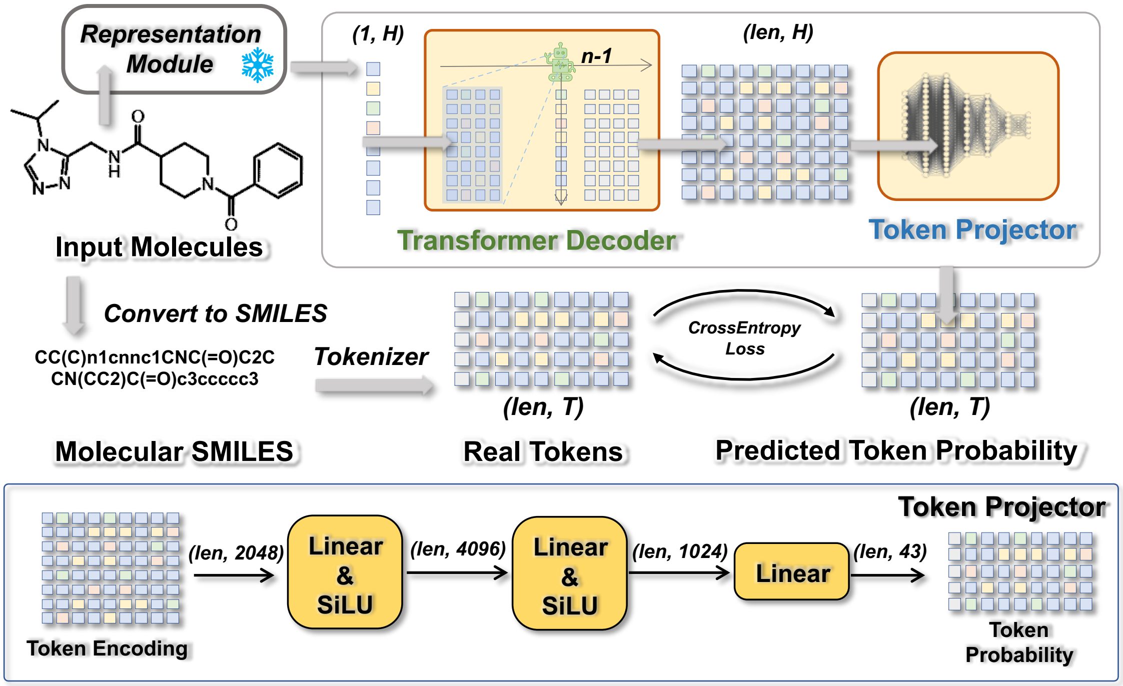

Figure S3 | Training strategies for the reconstruction module. Learnable parameters are boxed in brownish red, with 'H' referring to hidden size and 'T' to token size, 'len' to sequence length of padded SMILES in the batch. The tokenization strategy for SMILES is detailed in the **Methods** section of the main text. Token projector was used in reconstruction module.

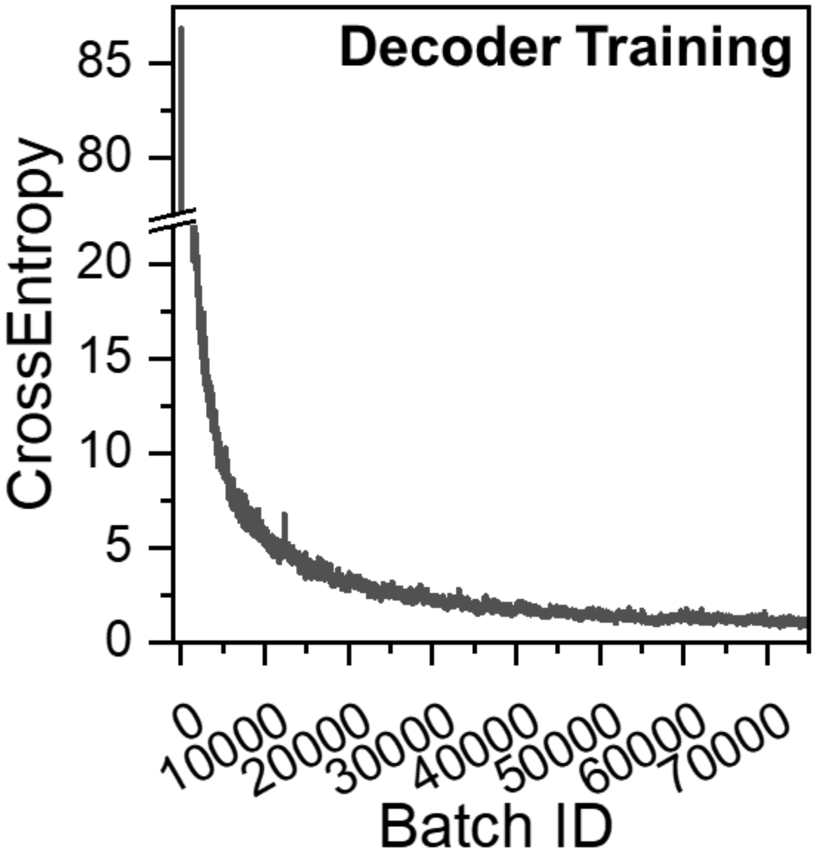

Figure S4 | The cross-entropy loss curve during training of the reconstruction module. The loss value recorded every 10 steps.

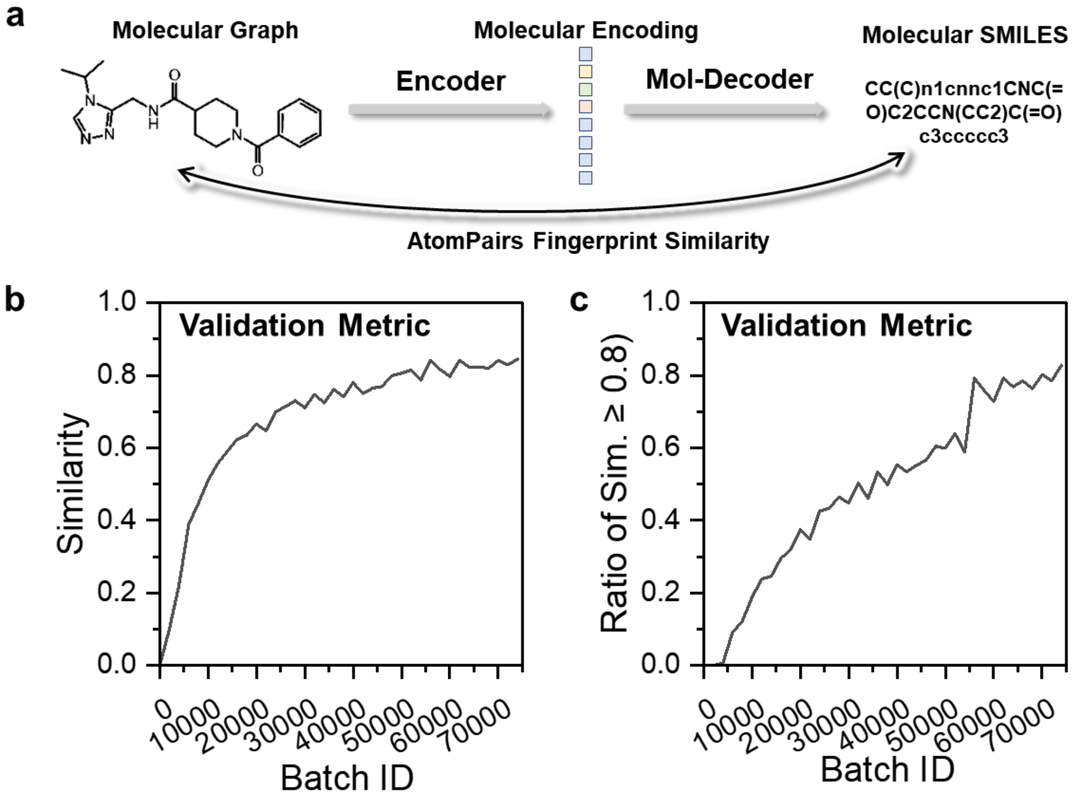

Figure S5 | The reconstruction of molecular structure for the validation set during training stage of Ouroboros. (a) The validation scheme for molecular decoder. The performance of the molecular decoder was evaluated by calculating the AtomPairs molecular fingerprint similarity (MFS) between the decoded and the original molecular structures. **(b)** The similarity curve of the validation set during training. The model was validated every 2000 steps. **(c)** As the model converges, the percentage of molecules that successfully recover the original structure (defined as a similarity greater than 0.8) gradually increases.

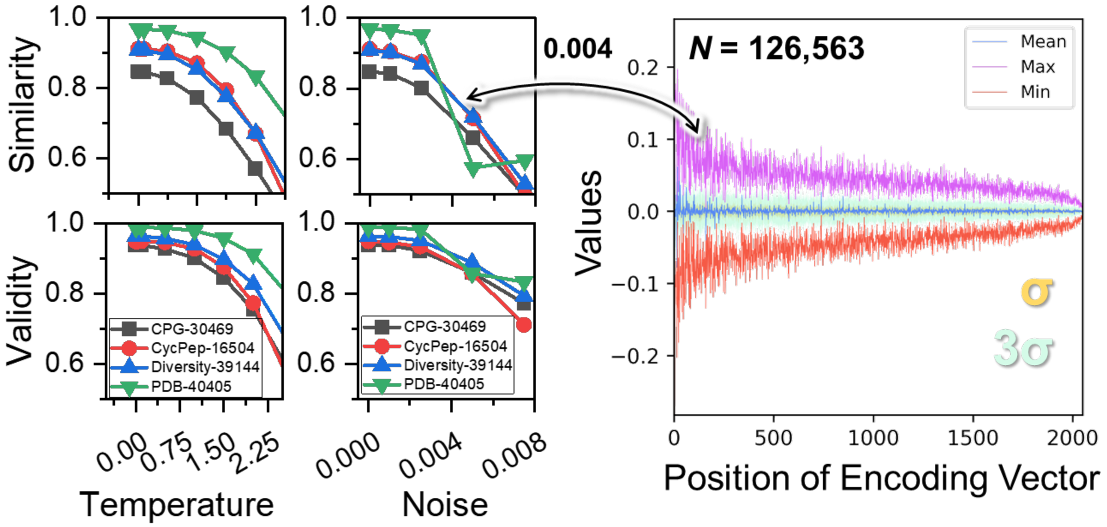

Figure S6 | The investigations of the Ouroboros exploration capability onto neighborhood representation space. The temperature affects the sampling of tokens by the decoder, implemented as a Gumbel-max trick. Noise will be randomly added to 50% of the positions in the 1D encoding vector with a Gaussian distribution. The value interval of the noise is determined based on the distribution of the encoded vectors, and for the encoded vectors generated by the representation module, the maximum, minimum, mean, σ (standard deviation) and 3σ at each position are shown on the right. The temperature is varied in the interval between 0 and 2, and the noise is varied in the interval between 0 and 0.0075.

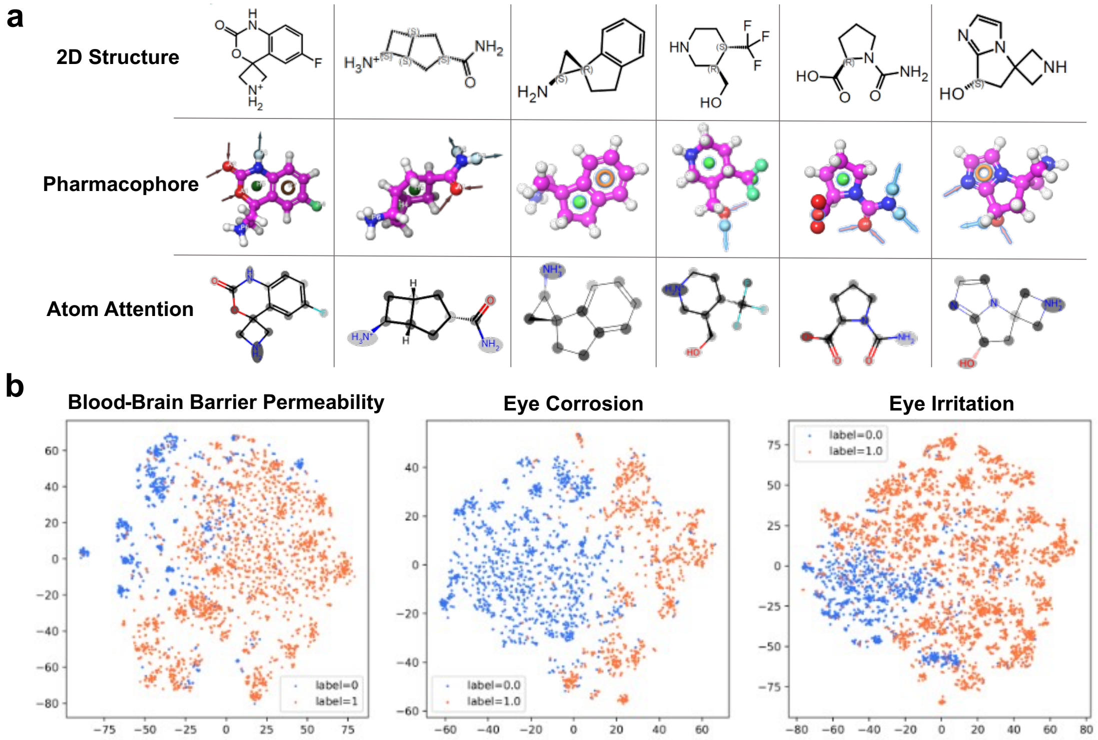

Figure S7 | Exploring chemical information in the atom weights and 2048-dimensional representation vector of Ouroboros. (a) Visualization of global self-attention mechanism in representation module. The attentional weights of the different atoms are indicated by the shade of the background color, suggesting that the representation module is able to sense the interactions between the different functional groups within molecular structure. **(b)** The t-SNE dimensionality reduction analysis revealed chemical features of the compounds related to conformation, formal charge, and acidity/basicity.

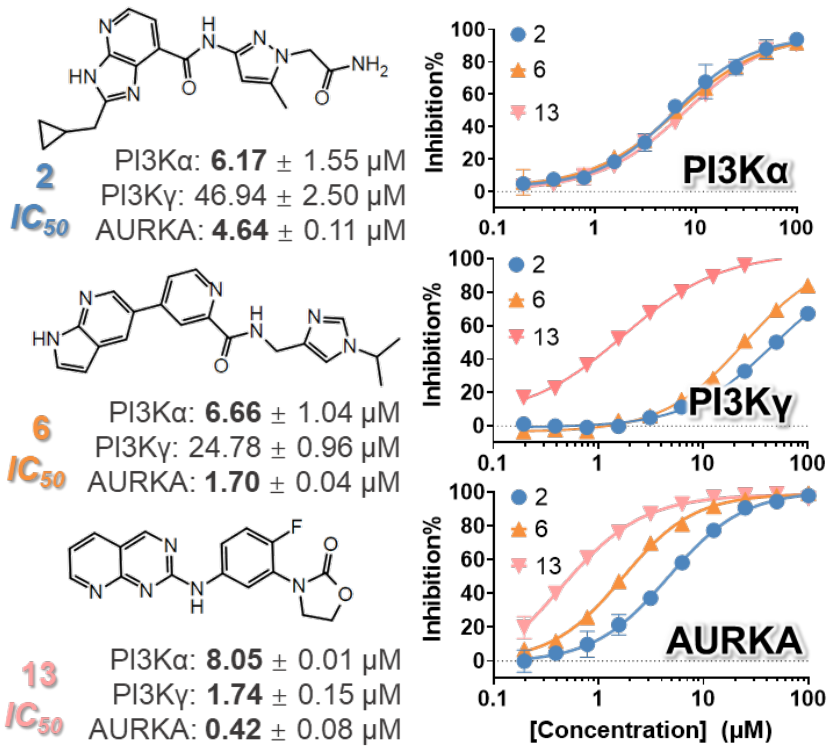

Figure S8 | Inhibition curves and *IC₅₀* of three hit compounds.

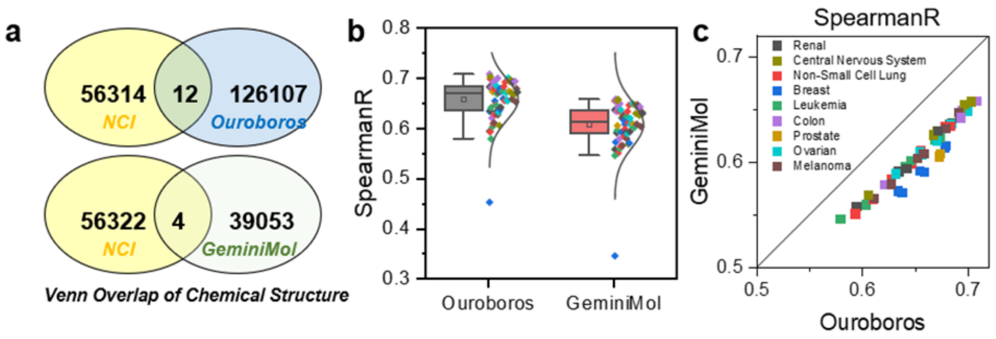

Figure S9 | Head-to-Head Comparison of Ouroboros and GeminiMol on the NCI Dataset. a) The chemical overlap between NCI datasets with molecular datasets of Ouroboros/GeminiMol. **b)** The comparison of overall performance of Ouroboros and GeminiMol. **c)** The head-to-head comparison between Ouroboros and GeminiMol. Different source of cancer cell lines was colored in different colors.

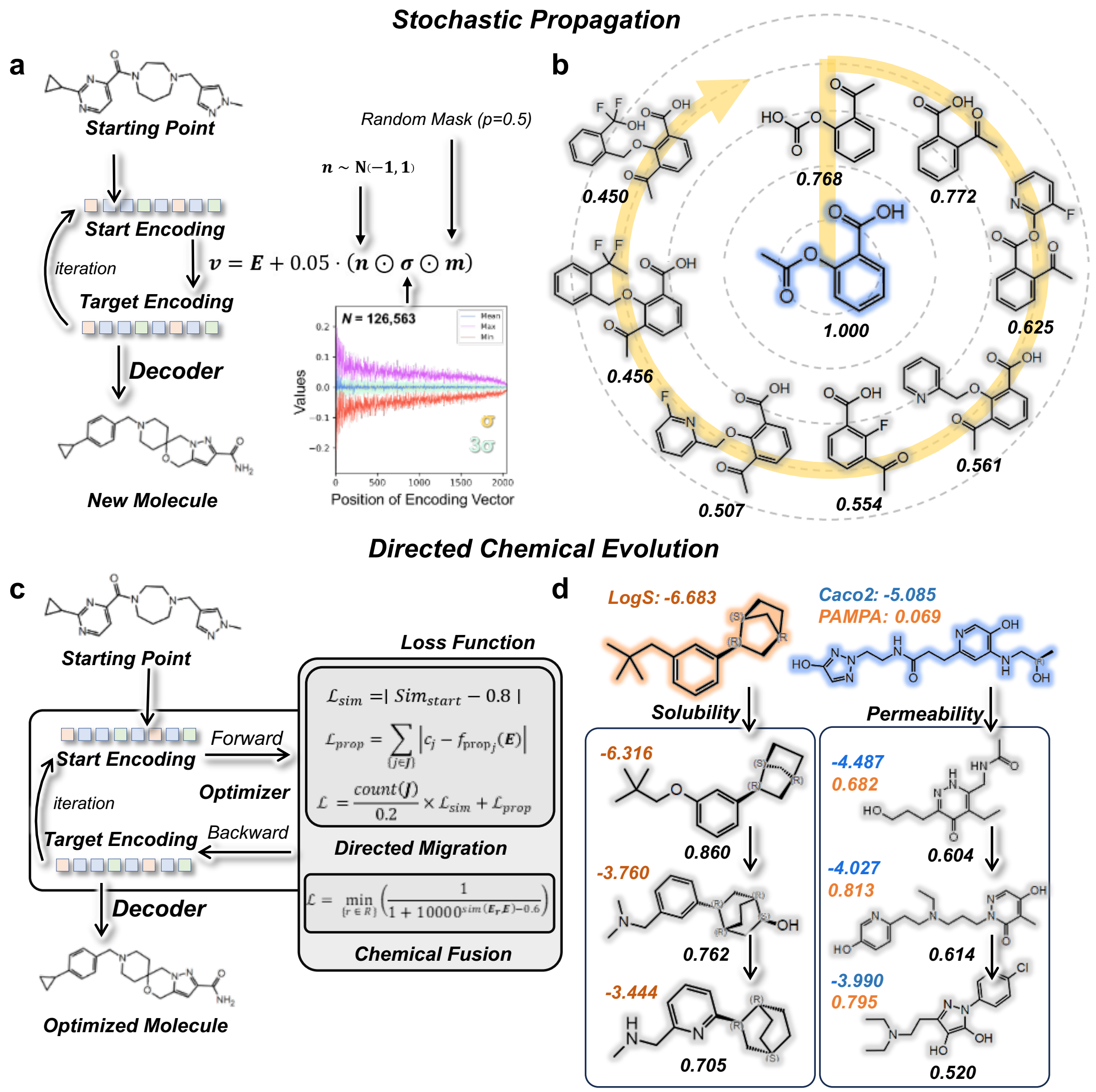

Figure S10 | Exploring chemical encoding space with Ouroboros. (a) Perturbation of the compound structure is implemented by adding Gaussian noise to the encoding vector. **(b)** Stochastic propagation from aspirin. Encoding similarities with start molecule are marked at the bottom of molecules. **(c)** Directed chemical evolution by backward propagation through the optimizer. The different loss functions were used in directed migration and chemical fusion. **(d)** Directed migration for optimizing solubility and membrane permeability. The property labels are predictions from the property predictor trained during the benchmark test. The encoding similarity values are displayed beneath each molecule. Solubility is represented by LogS (shown in brown, positioned to the left of each molecule), while membrane permeability is indicated by LogPeff (measured in 10^{-6} cm/s). Parallel artificial membrane permeability values are highlighted in orange, and Caco-2 cell permeability values are shown in blue. PAMPA (Parallel Artificial Membrane Permeability Assay) is also included as a reference.

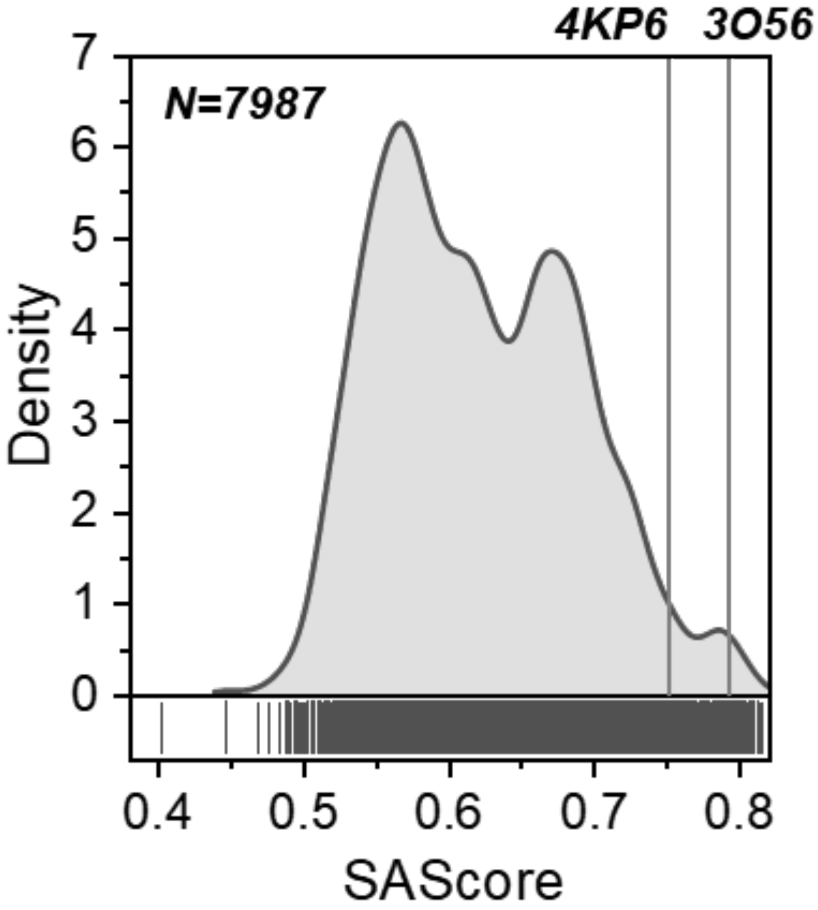

Figure S11 | Distribution of SA scores for molecules generated during directed migration. The SA scores for the start and target molecules are displayed as vertical reference lines.

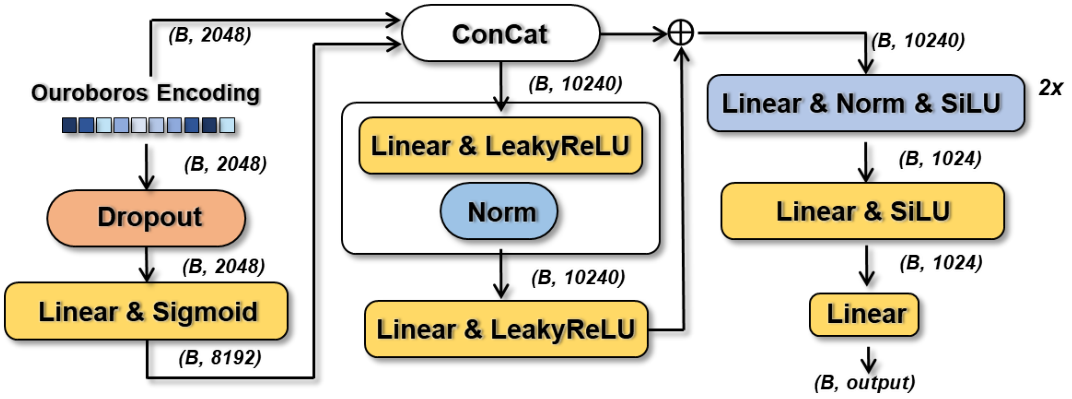

Figure S12 | The implementation of property predictor for molecular proptery modeling. The ‘B’ refers to batch size.

Supplementary Tables

Table S1 | Performance comparison of Ouroboros on validation and test sets in similarity learning

	**RMSE**		**PEARSONR**		**SPEARMANR**	
	Validation	Test	Validation	Test	Validation	Test
ECFP4 Tanimoto	0.016	0.016	0.890	0.878	0.883	0.874
AtomPairs Tversky	0.029	0.028	0.949	0.959	0.940	0.954
MACCS Tanimoto	0.023	0.023	0.983	0.979	0.983	0.977
CSS_0.14806	0.046	0.035	0.933	0.953	0.946	0.955
CSS_0.5060	0.044	0.033	0.938	0.958	0.950	0.960
CSS_0.88836	0.043	0.033	0.940	0.960	0.954	0.962
CSS_1.4806	0.043	0.033	0.941	0.960	0.955	0.962

Table S2 | Virtual screening benchmark results on DUD-E and LIT-PCBA*

Methods	Pre-Training Data Size	AUPRC	AUROC	BEDROC[1]	EF0.1%	EF1.0%	logAUC
Performance on DUD-E[2]							
Ouroboros	120 K	<u>0.363</u>	0.777	<u>0.557</u>	<u>29.925</u>	20.272	<u>0.472</u>
GeminiMol	39 K	0.307	0.734	0.490	27.227	17.718	0.418
ECFP4	/	0.345	0.753	0.556	**30.230**	<u>20.360</u>	0.450
AtomPairs	/	0.278	0.752	0.469	27.656	16.469	0.408
MACCS	/	0.230	0.720	0.380	22.792	13.289	0.365
MolT5	100 M (ZINC)	0.247	0.742	0.417	24.879	14.239	0.383
MolFormer	1.1 B[3] (ZINC+PubChem)	**0.386**	**0.836**	**0.569**	29.803	**20.675**	**0.509**
MolMetaLM	110 M (PubChem)	0.118	0.640	0.210	15.778	6.286	0.258
ChemBERTa	100 K (ZINC)	0.202	0.679	0.351	22.079	11.969	0.330
ChemMLM[4]	77 M (PubChem)	0.295	<u>0.782</u>	0.476	26.681	16.961	0.431
ChemMTR[4]	77 M (PubChem)	0.239	0.686	0.415	24.030	14.678	0.363
Performance on LIT-PCBA[2]							
Ouroboros	120 K	**0.030**	0.579	**0.078**	**24.925**	**7.575**	<u>0.222</u>
GeminiMol	39 K	<u>0.029</u>	<u>0.592</u>	<u>0.074</u>	<u>24.022</u>	<u>6.559</u>	<u>0.222</u>
ECFP4	/	0.021	0.527	0.053	16.361	4.483	0.189
AtomPairs	/	0.024	0.561	0.058	22.368	5.809	0.206
MACCS	/	0.017	0.557	0.043	10.752	3.891	0.196
MolT5	100 M (ZINC)	0.014	0.616	0.040	16.481	4.009	0.211
MolFormer	1.1 B[3] (ZINC+PubChem)	<u>0.029</u>	**0.646**	0.061	19.787	6.046	**0.245**
MolMetaLM	110 M (PubChem)	0.012	0.575	0.027	6.975	2.533	0.187
ChemBERTa	100 K (ZINC)	0.015	0.575	0.042	15.910	4.771	0.197
ChemMLM[4]	77 M (PubChem)	0.024	0.579	0.058	17.698	6.038	0.207
ChemMTR[4]	77 M (PubChem)	0.021	0.508	0.043	19.073	4.672	0.175

*Performance for the best model for each metric are bolded. The second ranked method is indicated by an underline.

[1] BEDROC is calculated under the α set to 160.9.

[2] Part of the decoys for DUD-E collected from ZINC, and all molecules for LIT-PCBA collected from PubChem. These two molecular databases are common sources for molecular representation learning.

[3] Open-sourced MolFormer was only trained on 10% of the data from ZINC and PubChem, which is approximately 0.11 billion.

[4] Two variants of ChemBERTa-2.

Table S3 | Inhibitory activity of 18 candidate compounds on 7 kinase targets*

**Compound ID #**	**AVE Inhibition% (10 μM)**						
	PIK3CA	PIK3CG	AURKA	MEK1	CHK1	PLK1	WEE1
1	19.03	-2.64	27.94	-7.70	3.66	-7.29	2.51
2	**60.18**	14.77	**73.57**	-12.02	4.87	**54.49**	15.76
3	11.86	30.81	**67.10**	-4.12	3.26	3.49	5.67
4	19.05	17.62	31.44	-5.99	10.08	-12.88	8.13
5	9.16	-2.68	31.20	-1.95	8.08	-1.30	7.67
6	**60.71**	29.76	**87.25**	10.14	25.38	-18.68	0.18
7	2.88	-2.08	22.31	-6.28	-0.69	-6.89	5.05
8	10.18	8.05	**58.96**	-7.64	1.48	-15.37	8.87
9	20.71	19.79	**52.94**	0.06	-0.49	-5.46	5.92
10	11.42	13.51	40.26	-20.06	-2.24	-22.99	6.84
11	36.66	23.65	**83.30**	12.20	3.54	-4.19	12.91
12	9.99	4.92	38.74	-21.91	-5.82	-17.73	-0.15
13	**52.82**	**86.07**	**96.00**	14.02	4.20	4.02	44.00
14	7.28	4.28	23.75	-3.36	7.02	-2.35	-1.34
15	38.79	16.36	38.56	-3.02	-1.46	41.26	5.63
16	42.96	32.00	29.45	13.65	3.47	9.62	7.97
17	-1.05	4.28	32.46	-6.02	0.04	-7.03	7.42
18	23.76	9.31	12.50	-4.09	1.98	-7.67	2.87

* All data are repeated twice and the values in the table are averaged. Values with inhibitions above 50 % are colored and bolded in black.

Table S4 | Spearman correlation coefficients on the ADMET properties projection (frozen representation module)[*]

Property	Size	Ouroboros	GeminiMol	CombineFP	ECFP4	AtomPairs	MACCS	RDK
Caco2	818	<u>0.791</u>	**0.799**	0.755	0.732	0.685	0.619	0.535
Clearance Hepatocyte	1091	<u>0.500</u>	**0.506**	0.402	0.419	0.410	0.259	0.305
Clearance Microsome	992	**0.601**	<u>0.570</u>	0.513	0.562	0.463	0.475	0.483
Half Life	601	**0.537**	0.380	<u>0.431</u>	0.401	0.379	0.204	0.377
Hydration Free Energy	587	0.886	<u>0.897</u>	0.883	0.878	0.759	**0.913**	0.688
LD50	6641	**0.635**	0.537	0.629	0.573	<u>0.615</u>	0.563	0.550
Lipophilicity	3780	**0.816**	0.715	<u>0.780</u>	0.714	0.722	0.596	0.520
PPBR	1453	0.557	0.581	<u>0.630</u>	0.576	**0.641**	0.457	0.553
Solubility	8876	**0.833**	0.801	<u>0.814</u>	0.813	0.795	0.797	0.701
SSDV	1017	<u>0.540</u>	0.522	**0.571**	0.543	0.504	0.509	0.390
Mean	/	**0.670**	0.631	<u>0.641</u>	0.621	0.597	0.539	0.510

[*]Performance for the best model for each task are bolded. The second ranked method is indicated by an underline.

[1]Steady-State Distribution Volume

Table S5 | Spearman correlation coefficients on the ADMET properties fine-tuning*

Property	Size	Ouroboros	GeminiMol	Uni-Mol	ChemBERTa	MolT5	MolFormer	FP-GNN	MolMetaLM
Caco2	818	0.815	<u>0.821</u>	0.799	0.806	0.76	0.805	**0.843**	0.807
Clearance Hepatocyte	1091	0.405	0.418	0.223	**0.487**	0.372	0.404	0.399	<u>0.435</u>
Clearance Microsome	992	<u>0.618</u>	0.613	**0.625**	0.562	0.524	0.605	0.557	0.518
Half Life	601	<u>0.490</u>	0.409	**0.558**	0.267	-0.105	-0.223	0.338	0.226
Hydration Free Energy	587	0.905	<u>0.910</u>	**0.913**	0.873	0.847	0.891	0.845	0.892
LD50	6641	0.616	0.595	0.636	0.623	0.556	<u>0.663</u>	0.585	**0.678**
Lipophilicity	3780	**0.873**	0.844	<u>0.850</u>	0.802	0.775	0.823	0.790	0.836
PPBR	1453	**0.716**	<u>0.698</u>	0.617	0.583	0.542	0.645	0.637	0.561
Solubility	8876	0.848	0.832	**0.882**	0.864	0.85	0.866	0.835	<u>0.868</u>
SSDV[1]	1017	<u>0.584</u>	0.508	**0.657**	0.471	0.378	-0.088	0.300	0.569
Mean	/	**0.687**	0.665	<u>0.676</u>	0.634	0.550	0.539	0.613	0.639

*Performance for the best model for each task are bolded. The second ranked method is indicated by an underline.

[1]Steady-State Distribution Volume
